# Supplementary material for: Occurrence of Antimicrobial-Resistant Escherichia coli in Marine Mammals of the North and Baltic Seas: Sentinels for Human Health
Source: Antibiotics (Basel). 2022 Sep 14;11(9):1248. doi: 10.3390/antibiotics11091248 (PMC9495373; doi:10.3390/antibiotics11091248)
Supplement: Supplementary file 1 [file antibiotics-11-01248-s001.zip › Figures - suplementary material.pdf]

Supplementary Figures

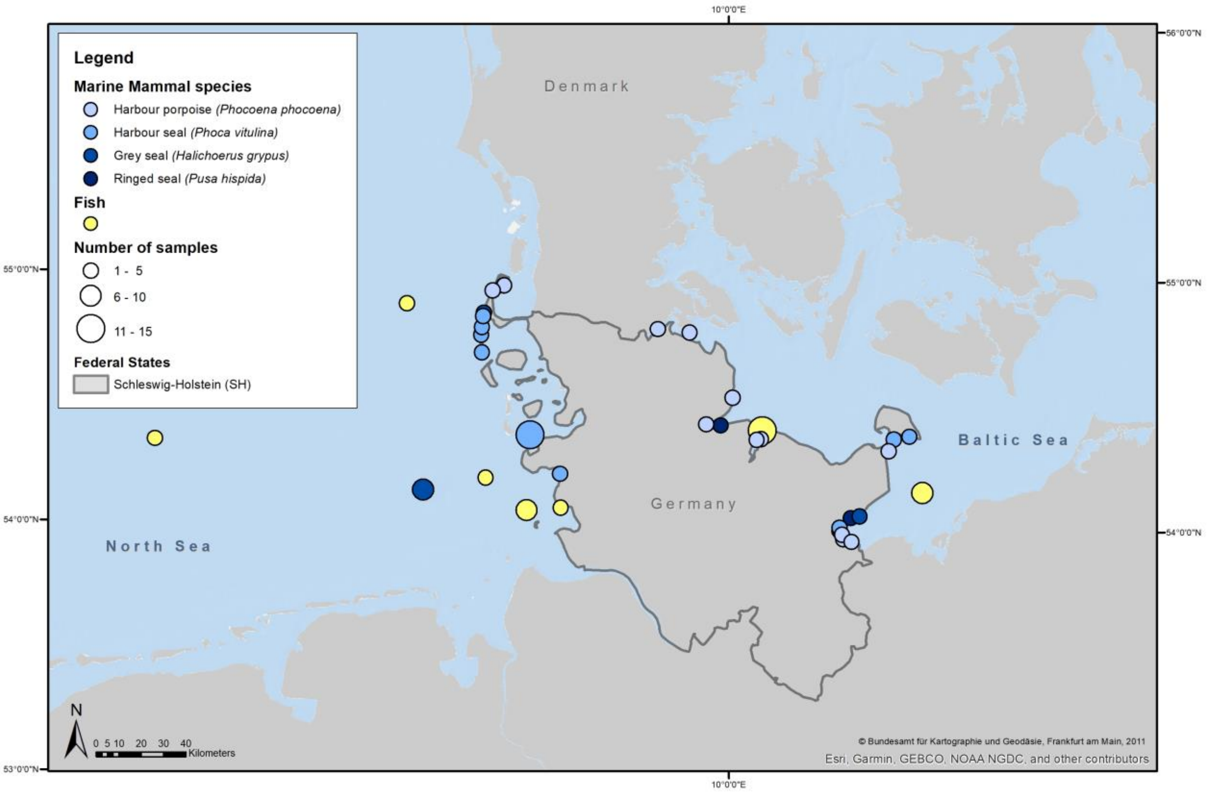

Figure S1: The map depicts the samples of fishes and marine mammals from the North and Baltic Sea coasts of Schleswig-Holstein, Germany.

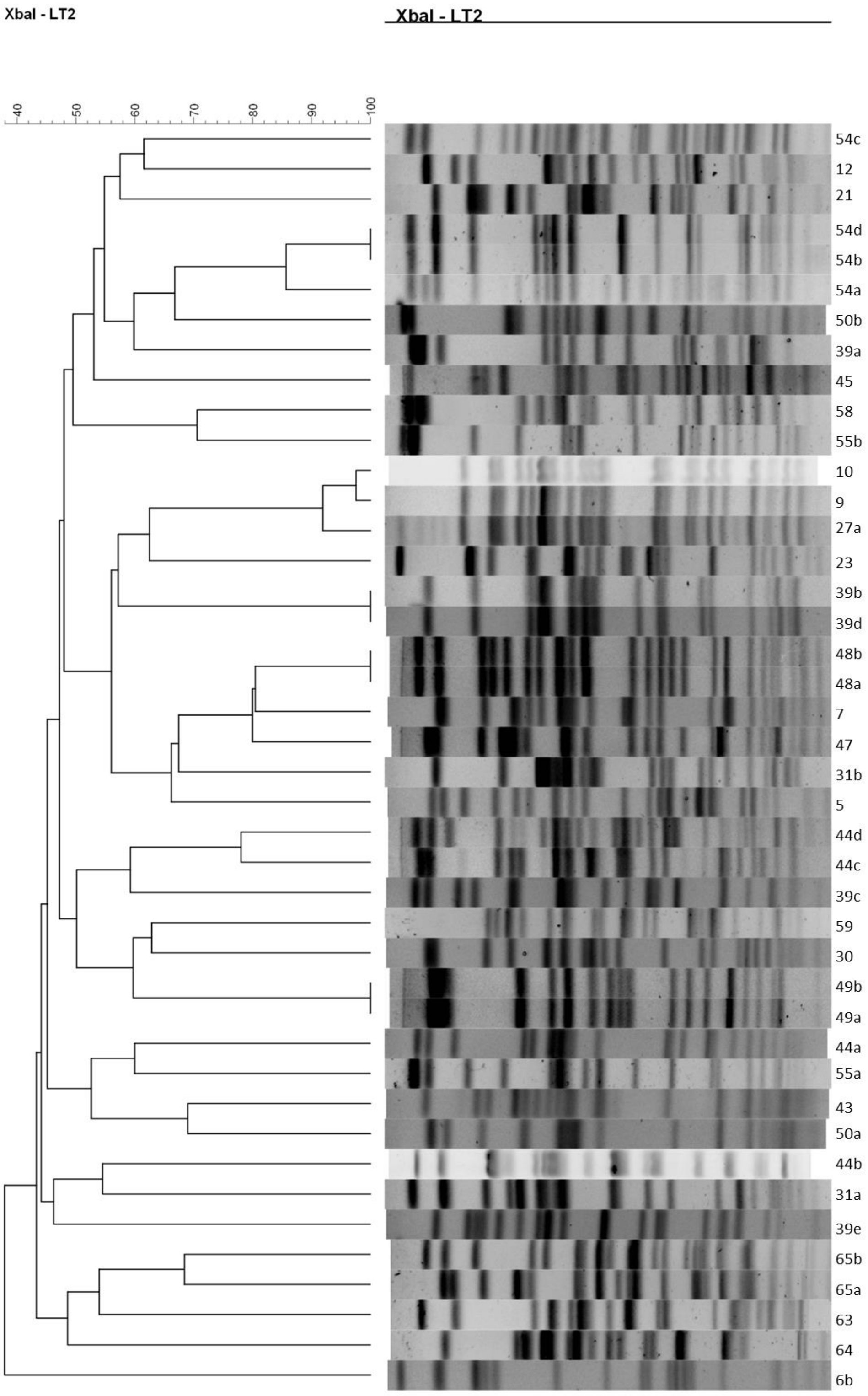

Figure S2: The figure shows the dendrogram including the pulsed-field gel electrophoresis band patterns of the 42 isolates that were typeable by XbaI macrorestriction.
