# Supplementary material for: Occurrence of Antimicrobial-Resistant Escherichia coli in Marine Mammals of the North and Baltic Seas: Sentinels for Human Health
Source: Antibiotics (Basel). 2022 Sep 14;11(9):1248. doi: 10.3390/antibiotics11091248 (PMC9495373; doi:10.3390/antibiotics11091248)
Supplement: Supplementary file 1 [file antibiotics-11-01248-s001.zip › Table S1_with change.pdf]

ID	Species	Location	Sea	Sampling date	Sample method	Age class	Sex	Nec-ropsy
1	Harbour seal (*Phoca vitulina*)	Sylt, DE	NS	31.03.2017	rectal - death	adult	m	yes
2	Grey seal (*Halichoerus grypus*)	Helgoland, DE	NS	30.03.2017	faecal - alive	n.d.	n.d.	no
3	Grey seal (*Halichoerus grypus*)	Helgoland, DE	NS	30.03.2017	faecal - alive	n.d.	n.d.	no
4	Grey seal (*Halichoerus grypus*)	Helgoland, DE	NS	30.03.2017	faecal - alive	n.d.	n.d.	no
5	Grey seal (*Halichoerus grypus*)	Helgoland, DE	NS	30.03.2017	faecal - alive	n.d.	n.d.	no
6	Grey seal (*Halichoerus grypus*)	Helgoland, DE	NS	30.03.2017	faecal - alive	n.d.	n.d.	no
7	Grey seal (*Halichoerus grypus*)	Helgoland, DE	NS	30.03.2017	faecal - alive	n.d.	n.d.	no
8	Harbour seal (*Phoca vitulina*)	Husum, DE	NS	19.04.2017	rectal - alive	adult	f	no
9	Harbour seal (*Phoca vitulina*)	Husum, DE	NS	19.04.2017	rectal - alive	adult	f	no
10	Harbour seal (*Phoca vitulina*)	Husum, DE	NS	19.04.2017	rectal - alive	adult	m	no
11	Harbour seal (*Phoca vitulina*)	Husum, DE	NS	19.04.2017	rectal - alive	adult	m	no
12	Harbour seal (*Phoca vitulina*)	Husum, DE	NS	19.04.2017	rectal - alive	adult	f	no
13	Harbour seal (*Phoca vitulina*)	Husum, DE	NS	19.04.2017	rectal - alive	adult	m	no
14	Harbour seal (*Phoca vitulina*)	Husum, DE	NS	19.04.2017	rectal - alive	adult	m	no
15	Harbour seal (*Phoca vitulina*)	Husum, DE	NS	19.04.2017	rectal - alive	adult	m	no
16	Harbour seal (*Phoca vitulina*)	Husum, DE	NS	19.04.2017	rectal - alive	adult	m	no
17	Harbour seal (*Phoca vitulina*)	Husum, DE	NS	19.04.2017	rectal - alive	adult	m	no
18	Harbour seal (*Phoca vitulina*)	Husum, DE	NS	19.04.2017	rectal - alive	adult	f	no
19	Harbour seal (*Phoca vitulina*)	Husum, DE	NS	19.04.2017	rectal - alive	adult	f	no
20	Harbour seal (*Phoca vitulina*)	Husum, DE	NS	19.04.2017	rectal - alive	adult	m	no
21	Harbour seal (*Phoca vitulina*)	Husum, DE	NS	19.04.2017	rectal - alive	adult	m	no
22	Harbour seal (*Phoca vitulina*)	Husum, DE	NS	19.04.2017	rectal - alive	adult	m	no
23	Harbour seal (*Phoca vitulina*)	Bay of Lübeck, DE	BS	28.05.2017	rectal - death	juvenile	m	yes
24	Harbour seal (*Phoca vitulina*)	Sylt, DE	NS	18.04.2017	rectal - death	juvenile	f	yes
25	Harbour seal (*Phoca vitulina*)	Sylt, DE	NS	24.04.2017	rectal - death	juvenile	f	yes
26	Harbour seal (*Phoca vitulina*)	Sylt, DE	NS	07.05.2017	rectal - death	juvenile	m	yes
27	Harbour seal (*Phoca vitulina*)	Sylt, DE	NS	14.05.2017	rectal - death	juvenile	f	yes
28	Grey seal (*Halichoerus grypus*)	Sylt, DE	NS	20.05.2017	rectal - death	juvenile	f	yes
29	Harbour porpoise (*Phocoena phocoena*)	Sylt, DE	NS	10.06.2017	rectal - death	neonate	f	yes

30	Harbour porpoise (*Phocoena phocoena*)	Sylt, DE	NS	10.06.2017	rectal - death	juvenile	m	yes
31	Harbour seal (*Phoca vitulina*)	Eider Barrage, DE	NS	16.06.2017	rectal - death	neonate	f	yes
32	Harbour porpoise (*Phocoena phocoena*)	Bay of Lübeck, DE	BS	28.06.2017	rectal - death	juvenile	f	yes
33	Harbour porpoise (*Phocoena phocoena*)	Bay of Kiel, DE	BS	20.05.2017	rectal - death	adult	m	yes
34	Harbour porpoise (*Phocoena phocoena*)	Bay of Kiel, DE	BS	05.08.2017	rectal - death	juvenile	f	yes
35	Harbour porpoise (*Phocoena phocoena*)	Bay of Kiel, DE	BS	29.08.2017	rectal - death	juvenile	m	yes
36	Harbour porpoise (*Phocoena phocoena*)	Bay of Kiel, DE	BS	04.11.2017	rectal - death	juvenile	f	yes
37	Harbour porpoise (*Phocoena phocoena*)	Bay of Kiel, DE	BS	16.11.2017	rectal - death	adult	m	yes
38	Grey seal (*Halichoerus grypus*)	Bay of Lübeck, DE	BS	12.01.2018	faecal - alive	adult	n.d.	no
39	Ringed seal (*Pusa hispida)*	Bay of Lübeck, DE	BS	22.01.2018	rectal - death	juvenile	f	yes
40	Grey seal (*Halichoerus grypus*)	Bay of Lübeck, DE	BS	28.05.2018	rectal - death	juvenile	m	yes
41	Harbour porpoise (*Phocoena phocoena*)	Kattegat, DK	BS	23.05.2018	rectal - alive	juvenile	m	no
42	Harbour porpoise (*Phocoena phocoena*)	Kattegat, DK	BS	23.05.2018	rectal - alive	juvenile	m	no
43	Harbour porpoise (*Phocoena phocoena*)	Bay of Lübeck, DE	BS	24.05.2018	rectal - death	adult	f	yes
44	Harbour porpoise (*Phocoena phocoena*)	Bay of Kiel, DE	BS	02.08.2018	rectal - death	neonate	m	yes
45	Ringed seal (*Pusa hispida*)	Bay of Kiel, DE	BS	02.09.2018	rectal - death	juvenile	m	yes
46	Ringed seal (*Pusa hispida*)	Bothnian Bay, SE	BS	12.06.2018	rectal - hunted	juvenile	f	yes
47	Ringed seal (*Pusa hispida*)	Bothnian Bay, SE	BS	13.06.2018	rectal - hunted	juvenile	m	yes
48	Ringed seal (*Pusa hispida*)	Bothnian Bay, SE	BS	14.06.2018	rectal - hunted	juvenile	m	yes
49	Ringed seal (*Pusa hispida*)	Bothnian Bay, SE	BS	14.06.2018	rectal - hunted	adult	f	yes
50	Harbour seal (*Phoca vitulina*)	Bay of Kiel, DE	BS	23.08.2018	rectal - death	juvenile	f	yes
51	Harbour porpoise (*Phocoena phocoena*)	Bay of Kiel, DE	BS	20.08.2018	rectal - death	neonate	m	yes
52	Harbour porpoise (*Phocoena phocoena*)	Bay of Lübeck, DE	BS	03.08.2018	rectal - death	neonate	m	yes
53	Harbour porpoise (*Phocoena phocoena*)	Bay of Lübeck, DE	BS	17.11.2018	rectal - death	adult	m	yes
54	Harbour porpoise (*Phocoena phocoena*)	Bay of Lübeck, DE	BS	01.12.2018	rectal - death	adult	m	yes
55	Harbour seal (*Phoca vitulina*)	Bay of Mecklenburg, DE	BS	01.11.2018	rectal - death	juvenile	m	yes
56	Grey seal (*Halichoerus grypus*)	Bay of Lübeck, DE	BS	10.01.2019	faecal - alive	n.d.	n.d.	no
57	Ringed seal (*Pusa hispida*)	Bothnian Bay, SE	BS	04.06.2019	rectal - hunted	juvenile	m	yes
58	Ringed seal (*Pusa hispida*)	Bothnian Bay, SE	BS	05.06.2019	rectal - hunted	juvenile	f	yes
59	Ringed seal (*Pusa hispida*)	Bothnian Bay, SE	BS	05.06.2019	rectal - hunted	adult	m	yes
60	Ringed seal (*Pusa hispida*)	Bothnian Bay, SE	BS	05.06.2019	rectal - hunted	adult	m	yes

61	Ringed seal (*Pusa hispida*)	Bothnian Bay, SE	BS	06.06.2019	rectal - hunted	adult	f	yes
62	Ringed seal (*Pusa hispida*)	Bothnian Bay, SE	BS	07.06.2019	rectal - hunted	juvenile	f	yes
63	Ringed seal (*Pusa hispida*)	Bothnian Bay, SE	BS	07.06.2019	rectal - hunted	adult	f	yes
64	Ringed seal (*Pusa hispida*)	Bothnian Bay, SE	BS	07.06.2019	rectal - hunted	adult	f	yes
65	Ringed seal (*Pusa hispida*)	Bothnian Bay, SE	BS	07.06.2019	rectal - hunted	adult	m	yes
66	Ringed seal (*Pusa hispida*)	Bothnian Bay, SE	BS	08.06.2019	rectal - hunted	juvenile	f	yes
67	Atlantic herring (*Clupea harengus*)	Bay of Mecklenburg, DE	BS	26.11.2017	rectal - death	n.d.	f	no
68	Atlantic herring (*Clupea harengus*)	Bay of Mecklenburg, DE	BS	26.11.2017	rectal - death	n.d.	m	no
69	Plaice (*Pleuronectes platessa*)	Bay of Mecklenburg, DE	BS	26.11.2017	rectal - death	n.d.	f	no
70	Plaice (*Pleuronectes platessa*)	Bay of Mecklenburg, DE	BS	26.11.2017	rectal - death	n.d.	m	no
71	Atlantic cod (*Gadus morhua*)	Bay of Mecklenburg, DE	BS	26.11.2017	rectal - death	n.d.	n.d.	no
72	Atlantic cod (*Gadus morhua*)	Bay of Mecklenburg, DE	BS	26.11.2017	rectal - death	n.d.	n.d.	no
73	Atlantic cod (*Gadus morhua*)	Bay of Meckelnburg, DE	BS	26.11.2017	rectal - death	n.d.	n.d.	no
74	Common dab (*Limanda limanda*)	Bay of Kiel, DE	BS	02.03.2018	rectal - death	n.d.	f	no
75	Common dab (*Limanda limanda*)	Bay of Kiel, DE	BS	02.03.2018	rectal - death	n.d.	f	no
76	Common dab (*Limanda limanda*)	Bay of Kiel, DE	BS	02.03.2018	rectal - death	n.d.	f	no
77	Common dab (*Limanda limanda*)	Bay of Kiel, DE	BS	02.03.2018	rectal - death	n.d.	f	no
78	Common dab (*Limanda limanda*)	Bay of Kiel, DE	BS	02.03.2018	rectal - death	n.d.	f	no
79	Common dab (*Limanda limanda*)	Bay of Kiel, DE	BS	02.03.2018	rectal - death	n.d.	f	no
80	Common dab (*Limanda limanda*)	Bay of Kiel, DE	BS	02.03.2018	rectal - death	n.d.	f	no
81	European flounder (*Platichthys flesus*)	Bay of Kiel, DE	BS	02.03.2018	rectal - death	n.d.	f	no
82	European flounder (*Platichthys flesus*)	Bay of Kiel, DE	BS	02.03.2018	rectal - death	n.d.	m	no
83	European flounder (*Platichthys flesus*)	Bay of Kiel, DE	BS	02.03.2018	rectal - death	n.d.	m	no
84	Plaice (*Pleuronectes platessa*)	Bay of Kiel, DE	BS	02.03.2018	rectal - death	n.d.	f	no
85	Plaice (*Pleuronectes platessa*)	Bay of Kiel, DE	BS	02.03.2018	rectal - death	n.d.	f	no
86	Plaice (*Pleuronectes platessa*)	Bay of Kiel, DE	BS	02.03.2018	rectal - death	n.d.	m	no
87	Atlantic mackerel (*Scomber scombrus*)	German waddensea	NS	20.07.2018	rectal - death	n.d.	n.d.	no
88	Atlantic mackerel (*Scomber scombrus*)	German waddensea	NS	20.07.2018	rectal - death	n.d.	n.d.	no
89	Atlantic mackerel (*Scomber scombrus*)	German waddensea	NS	20.07.2018	rectal - death	n.d.	n.d.	no
90	Atlantic mackerel (*Scomber scombrus*)	German waddensea	NS	20.07.2018	rectal - death	n.d.	n.d.	no

91	Atlantic mackerel (*Scomber scombrus*)	German waddensea	NS	20.07.2018	rectal - death	n.d.	n.d.	no
92	European smelt (*Osmerus eperlanus*)	German waddensea	NS	16.08.2018	rectal - death	n.d.	n.d.	no
93	European smelt (*Osmerus eperlanus*)	German waddensea	NS	16.08.2018	rectal - death	n.d.	n.d.	no
94	European smelt (*Osmerus eperlanus*)	German waddensea	NS	16.08.2018	rectal - death	n.d.	n.d.	no
95	European smelt (*Osmerus eperlanus*)	German waddensea	NS	16.08.2018	rectal - death	n.d.	n.d.	no
96	Plaice (*Pleuronectes platessa*)	German waddensea	NS	16.08.2018	rectal - death	n.d.	n.d.	no
97	Plaice (*Pleuronectes platessa*)	German waddensea	NS	16.08.2018	rectal - death	n.d.	n.d.	no
98	Solenette (*Buglossidium luteum*)	German waddensea	NS	16.08.2018	rectal - death	n.d.	n.d.	no
99	European Sea Sturgeon (*Acipenser sturio*)	German waddensea	NS	26.09.2018	rectal - death	n.d.	n.d.	no
100	Common dab (*Limanda limanda*)	North Sea	NS	09.07.2019	rectal - death	n.d.	f	no
101	Common dab (*Limanda limanda*)	North Sea	NS	09.07.2019	rectal - death	n.d.	m	no
102	Grey gurnard (*Eutrigla gurnardus*)	North Sea	NS	10.07.2019	rectal - death	n.d.	n.d.	no
103	Atlantic herring (*Clupea harengus*)	North Sea	NS	12.07.2019	rectal - death	n.d.	n.d.	no
104	Atlantic mackerel (*Scomber scombrus*)	North Sea	NS	13.07.2019	rectal - death	n.d.	n.d.	no
105	European pilchard (*Sardina pilchardus*)	North Sea	NS	13.07.2019	rectal - death	n.d.	n.d.	no
106	Whiting (*Merlangius merlangus*)	North Sea	NS	15.07.2019	rectal - death	n.d.	n.d.	no

Table S1: Samples information. Listed are information for each sample including sample ID, species, sample location and sea (NS = North Sea, BS = Baltic Sea), sample method, age class, sex (m = male, f = female) and if a necropsy was performed.
