# Supplementary material for: Occurrence of Antimicrobial-Resistant Escherichia coli in Marine Mammals of the North and Baltic Seas: Sentinels for Human Health
Source: Antibiotics (Basel). 2022 Sep 14;11(9):1248. doi: 10.3390/antibiotics11091248 (PMC9495373; doi:10.3390/antibiotics11091248)
Supplement: Supplementary file 1 [file antibiotics-11-01248-s001.zip › Table S2.pdf]

Sample ID	Grwoth on AB plate	No. of AB plates with bacterial growth	Ampicillin	Cephalothin	Chloramphenicol	Ciprofloxcin	Colistin	Gentamycin	Sulfasoxazole	Tetracyline	E. coli isolated
1	no	0	no	no	no	no	no	no	no	no	x
2	yes	1	no	no	yes	no	no	no	no	no	no
3	yes	1	no	no	yes	no	no	no	no	no	no
4	yes	2	no	yes	yes	no	no	no	no	no	no
5	yes	3	no	yes	yes	no	no	yes	no	no	yes
6	yes	2	no	yes	no	no	yes	no	no	no	yes
7	yes	1	no	no	no	no	yes	no	no	no	yes
8	yes	4	yes	yes	yes	no	no	no	yes	no	no
9	yes	4	yes	yes	no	no	yes	no	yes	no	yes
10	yes	1	no	no	no	no	yes	no	no	no	yes
11	yes	2	no	yes	yes	no	no	no	no	no	no
12	yes	4	no	yes	yes	no	yes	no	yes	no	yes
13	yes	1	no	no	no	no	no	no	yes	no	no
14	yes	1	yes	no	no	no	no	no	no	no	no
15	no	0	no	no	no	no	no	no	no	no	x
16	yes	4	no	yes	yes	no	yes	no	yes	no	no
17	no	0	no	no	no	no	no	no	no	no	x
18	yes	5	no	yes	yes	yes	yes	no	yes	no	no
19	yes	6	yes	yes	yes	yes	yes	no	yes	no	no
21	yes	4	no	yes	yes	no	yes	no	yes	no	no
22	yes	2	no	no	no	no	yes	no	yes	no	yes
23	yes	3	no	yes	yes	no	no	no	yes	no	no
24	yes	1	no	no	yes	no	no	no	no	no	yes
20	yes	1	no	no	no	no	yes	no	no	no	no

25	yes	1	no	no	no	no	yes	no	no	no	no
26	no	0	no	no	no	no	no	no	no	no	x
27	yes	4	yes	no	yes	no	yes	no	yes	no	yes
28	no	0	no	no	no	no	no	no	no	no	x
29	yes	3	yes	yes	yes	no	no	no	no	no	no
30	yes	1	no	no	no	no	yes	no	no	no	yes
31	yes	7	yes	yes	yes	yes	yes	no	yes	yes	yes
32	yes	6	yes	yes	yes	yes	yes	no	no	yes	no
33	yes	4	yes	no	no	yes	yes	no	yes	no	no
34	no	0	no	no	no	no	no	no	no	no	x
35	yes	4	yes	yes	yes	no	yes	no	no	no	no
36	yes	5	yes	yes	yes	yes	yes	no	no	no	no
37	yes	3	no	no	yes	no	yes	no	no	yes	no
38	yes	4	no	yes	yes	no	yes	no	no	yes	no
39	yes	3	yes	yes	no	no	no	no	no	yes	no
40	no	0	no	no	no	no	no	no	no	no	x
41	no	0	no	no	no	no	no	no	no	no	x
42	no	0	no	no	no	no	no	no	no	no	x
43	yes	2	no	no	yes	yes	no	no	no	no	no
44	no	0	no	no	no	no	no	no	no	no	x
45	yes	6	yes	yes	yes	no	yes	no	yes	yes	no
46	yes	7	yes	yes	yes	yes	yes	no	yes	yes	yes
47	no	0	no	no	no	no	no	no	no	no	x
48	no	0	no	no	no	no	no	no	no	no	x
49	no	0	no	no	no	no	no	no	no	no	x
50	no	0	no	no	no	no	no	no	no	no	x
51	no	0	no	no	no	no	no	no	no	no	x
52	no	0	no	no	no	no	no	no	no	no	x
53	no	0	no	no	no	no	no	no	no	no	x
54	no	0	no	no	no	no	no	no	no	no	x

55	no	0	no	no	no	no	no	no	no	no	x
56	yes	4	yes	yes	no	yes	yes	no	no	no	no
57	no	0	no	no	no	no	no	no	no	no	x
58	no	0	no	no	no	no	no	no	no	no	x
59	no	0	no	no	no	no	no	no	no	no	x
60	yes	5	yes	yes	yes	no	yes	no	no	yes	no
61	yes	5	yes	yes	no	no	yes	no	yes	yes	no
62	yes	1	no	yes	no	no	no	no	no	no	no
63	yes	6	yes	yes	yes	no	yes	no	yes	yes	yes
64	no	0	no	no	no	no	no	no	no	no	x
65	no	0	no	no	no	no	no	no	no	no	x
66	no	0	no	no	no	no	no	no	no	no	x
67	no	0	no	no	no	no	no	no	no	no	x
68	no	0	no	no	no	no	no	no	no	no	x
69	yes	7	yes	yes	yes	yes	yes	no	yes	yes	yes
70	yes	6	yes	yes	yes	yes	yes	no	yes	no	no
71	yes	6	yes	yes	yes	yes	yes	no	yes	no	no
72	yes	5	yes	yes	no	yes	yes	no	yes	no	no
73	yes	5	yes	yes	yes	no	yes	no	yes	no	no
76	yes	3	no	yes	no	no	yes	no	yes	no	no
77	yes	3	yes	yes	no	no	yes	no	no	no	no
78	yes	6	yes	yes	yes	yes	yes	no	yes	no	no
74	yes	6	yes	yes	yes	no	yes	yes	yes	no	yes
75	yes	1	no	no	no	no	yes	no	no	no	no
79	yes	3	no	no	yes	no	no	yes	yes	no	no
80	yes	4	no	yes	yes	no	yes	yes	no	no	yes
81	yes	6	yes	yes	yes	no	yes	yes	yes	no	yes
82	yes	4	no	yes	no	no	yes	yes	yes	no	yes
83	yes	7	yes	yes	yes	no	yes	yes	yes	yes	yes
84	yes	7	yes	yes	yes	yes	yes	no	yes	yes	no

85	yes	7	yes	yes	yes	yes	yes	no	yes	yes	no
86	yes	6	yes	yes	yes	yes	yes	no	no	yes	no
87	yes	8	yes	yes	yes	yes	yes	yes	yes	yes	yes
88	yes	6	yes	yes	yes	no	yes	no	yes	yes	yes
89	yes	6	yes	yes	yes	no	yes	yes	no	yes	no
90	no	0	no	no	no	no	no	no	no	no	no
91	yes	3	no	yes	yes	no	yes	no	no	no	yes
92	yes	1	no	no	no	no	yes	no	no	no	yes
93	no	0	no	no	no	no	no	no	no	no	x
94	yes	2	no	no	yes	no	yes	no	no	no	no
95	yes	3	yes	yes	no	no	yes	no	no	no	no
96	yes	2	no	yes	no	no	yes	no	no	no	yes
97	yes	3	yes	yes	no	no	yes	no	no	no	no
98	yes	4	yes	yes	no	no	yes	no	no	yes	yes
99	yes	2	yes	no	no	no	yes	no	no	no	no
100	yes	2	no	yes	yes	no	no	no	no	no	no
101	yes	4	yes	yes	yes	no	yes	no	no	no	no
102	no	0	no	no	no	no	no	no	no	no	x
103	yes	3	yes	yes	yes	no	no	no	no	no	no
104	no	0	no	no	no	no	no	no	no	no	x
105	yes	5	yes	yes	yes	yes	yes	no	no	no	no
106	no	0	no	no	no	no	no	no	no	no	x

Table S2: The table shows all marine mammal and fish samples and which sample grew on which antibiotic containing plate as well if *E. coli* was isolated from at least one of the plates. No *E. coli* isolation was performed in table cells with x because of missing bacterial growth on all antibiotic containing agar plates. All samples showed bacterial growth either on Blood agar, Gassner agar or in the Mossel bouillon indicating bacterial presence in the samples.
