# Supplementary material for: Occurrence of Antimicrobial-Resistant Escherichia coli in Marine Mammals of the North and Baltic Seas: Sentinels for Human Health
Source: Antibiotics (Basel). 2022 Sep 14;11(9):1248. doi: 10.3390/antibiotics11091248 (PMC9495373; doi:10.3390/antibiotics11091248)
Supplement: Supplementary file 1 [file antibiotics-11-01248-s001.zip › Table S3.pdf]

ID	Species	Resistance	Date of necropsy	Frozen	Main macroscopic findings	Parasitic burden	Microbiology
1	Pv	no	08.05.2017	yes	mild, acute, diffuse, alveolar bronchopneumonia; mild, chronic, focal, pyogranulomatous glossitis; mild, chronic, oligofocal, ulcerative gastritis; mild, acute, diffuse, interstitial lung emphysema; severe acute, diffuse, alveolar lung edema	stomach: mild infection; intestine: moderate infestation	not determined
56	Pv	yes	13.07.2017	yes	severe bronchopneumonia; moderate, acute, diffuse, alveolar oedema; suspicion of septicaemia	stomach: mild infection; small intestine: mild infection; large intestine; severe infection	lung: ++ coryneform bacteria, *Enterococcus faecalis*, non-fermenter; + *E. coli*, *Proteus* sp., yeasts; lung lymph node: ++ *Enterococcus faecalis*; + *E. coli*, a-haemolytic streptococci, non-fermenter, *Proteus* sp., yeasts, *Streptococcus dysgalactiae*; spleen: ++ *Acinetobacter* sp., non-fermenter; + *E. coli*, coryneform bacteria, *Enterococcus faecalis*, *Proteus* sp.; liver: ++ *Acinetobacter* sp., non-fermenter; + *Proteus* sp., *E. coli*, *Enterococcus faecalis*, yeasts, a-haemolytic streptococci; kidney: ++ *E. coli*, *Enterococcus faecalis*; + *Proteus* sp., *Enterococcus faecalis*, a-haemolytic streptococci, yeasts; intestine: + yeasts; mesenteric lymph node: + *E. coli*, yeasts; uterus: ++ *Enterococcus* sp.; + *E. coli*; *Pseudomonas* sp., *Proteus* sp., *Lactobacillus curvatus*; yeasts, *Streptococcus dysgalactiae*; brain: ++ *Enterococcus aquimarinus*; + a-haemolytic streptococci, *Acinetobacter Iwoffii*, *Proteus* sp., *Bacillus* sp., *E. coli*, non-fermenter

79	Pv	no	03.08.2017	yes	cachexia; icterus; moderate, subacute to chronic, diffuse, fibrinous-granulomatous bronchopneumonia; fatty liver; mild, diffuse, follicular hyperplasia of the spleen; severe, acute, diffuse alveolar and interstitial lung oedema; moderate, diffuse, acute, alveolar and interstitial lung oedema	lung: severe infection in bronchi and blood vessels; heart: mild infection in both ventricles; stomach: mild infection; intestine: mild infection in the small intestine and the caecum	lung: ++*Proteus* sp., *Pseudomonas fulva*, *Enterococcus faecalis*, *Vagococcus fluvialis*, *Bacillus* sp.; + *E. coli*, a-haemolytic streptococci, yeasts; kidney: ++ *Proteus* sp., *Pseudomonas fulva*, *Enterococcus faecalis*, *E. coli*, *Providencia rettgeri*; + *Enterobacter cloacae*, yeast; diaphragm: ++ *Enterococcus faecalis*, *Pseudomonas fulva*, a-haemolytic streptococci, non-fermenter; + *Bacillus* sp., *Proteus* sp., *E. coli*, yeasts; liver: ++ *Pseudomonas putida*, *Enterococcus* sp., non-fermenter, a-haemolytic streptococci; + *E. coli*, *Myroides* sp., yeasts; spleen: +++ *Acinetobacter* sp., a-haemolytic streptococci; ++ *E. coli*, *Enterococcus* sp., *Pseudomonas fulva*, *Pseudomonas putida*; + *Myroides* sp., *Proteus* sp.; intestine: ++ *E. coli*, + yeasts
80	Pv	yes	03.08.2017	yes	severe, acute, diffuse, alveolar pneumonia; mild, acute, diffuse hyperplasia of the lung lymph nodes; mild, acute diffuse hyperplasia of the tonsils; mild, chronic, focal, ulcerative gastritis; diarrhoea; mild, acute, diffuse, alveolar and interstitial lung emphysema; mild, acute, diffuse lung oedema	lung: moderate infection in bronchi and blood vessels; stomach: moderate infection; small intestine; moderate infection with acanthocephalan and cestodes	lung: +++ *Myroides odoratus*, *Streptococcus phocae*; ++ a-haemolytic streptococci, *E. coli*, *Serratia fonticola*; + *Enterococcus* sp., *Yarowia lipolytica*; liver: +++ *Streptococcus phocae*; ++ a-haemolytic streptococci, *Myroides odoratus*, *E. coli*; + *Enterococcus* sp., *Yarowia lipolytica*; intestine: ++ *Yarowia lipolytica*, *E. coli*; kidney: ++ *E. coli*, *Enterococcus faecalis*, a-haemolytic streptococci, *Streptococcus phocae*; + *Yarowia lipolytica*; spleen: +++ *Streptococcus phocae*, a-haemolytic streptococci; ++*E. coli*; + *Myroides* sp., *Bacillus* sp., *Yarowia lipolytica*
81	Pv	no	29.06.2017	yes	cachexia; moderate, subacute, diffuse, purulent pneumonia; moderate acute, diffuse, alveolar oedema	lung: severe infection in bronchi and blood vessels; stomach: mild infection; small intestine: moderate infection	not determined

82	Pv	yes	29.11.2017	yes	premature birth; subcutaneous haemorrhages at the head, neck and pelvis; diarrhoea; mild alveolar oedema	none	lung: + *Stenotrophomonas maltophilia*, yeasts, *Serratia marcescens*, *Enterococcus faecium*; liver: + *Stenotrophomonas maltophilia*, *E. coli*, *Bacillus* sp., yeasts; kidney: + *Cedecea* sp., *Enterococcus faecium*, *Stenotrophomonas maltophilia*, yeasts; spleen: ++ *Stenotrophomonas maltophilia*; + coagulase-negative Staphylococci, *E. coli*, yeasts; intestine: none
83	Hg	no	24.07.2017	yes	moderate, multifocal concrement deposition in and on the peritoneum of the abdominal wall, the intestine, the kidney and in the kidney; moderate haemo-hydro abdomen; mild, focal, ulcerative gastritis; mild haemo-hydro thorax and pericard; moderate hyperplasia of the pulpa of the spleen; multiple skin erosions on both hind flippers and the left abdominal wall; multiple loss of fur on the left abdominal wall; mild, diffuse, alveolar oedema; moderate, diffuse, alveolar and interstitial lung emphysema; suspicion of septicaemia	lung: mild infection in the bronchi; heart: moderate infection in the left ventricle; stomach and small intestine: moderate infection	liver: + *Pseudomonas fulva*, a-haemolytic streptococci, *Proteus* sp., *E. coli*, *Enterococcus* sp.; lung: + a-haemolytic streptococci, *Proteus* sp., *Acinetobacter johnsonii*, *E. coli*, *Enterococcus* sp.; spleen: + a-haemolytic streptococci, *Acinetobacter johnsonii*, *E. coli*, *Enterococcus* sp.; stomach ulcer: a-haemolytic streptococci, non-fermenter, yeasts, *E. coli*, *Enterococcus* sp.; brain: ++ a-haemolytic streptococci; + *Proteus* sp., *E. coli*, *Enterococcus* sp.; intestine: none; lung lymphnode: + a-haemolytic streptococci, non-fermenter, *E. coli*; uterus: ++ non-fermenter; a-haemolytic streptococci; + *Acinetobacter johnsonii*, *E. coli*, *Enterococcus* sp.
85	Pp	no	26.10.2017	yes	anaemia; moderate, acute, diffuse, alveolar lung oedema	none	brain: +++ non-fermenter, coagulase-negative staphylococci, α-haemolytic streptococci; ++ *E. coli*, *Citrobacter* sp., *Myroides* sp.; + yests, *Proteus* sp.; uterus: +++ *Rahnella aquatis*; ++ *Serratia* sp., *Enterococcus* sp., coagulase-negative staphylococci, *E. coli*, *Bacillus* sp.; + *Enterobacter cloacae*, *Myroides* sp.

86	Pp	yes	12.10.2017	yes	cachexia; moderate, acute, diffuse, alveolar bronchopneumonia; mild, chronic focal, ulcerative oesophagitis; mild, chronic, focal, pyogranulomatous steatitis (abscess close to the blow whole); severe, chronic, diffuse necrotic mesenterial lymphadenitis; severe, chronic, diffuse hyperplasia of the cortes of the adrenal glanda; severe, acute, diffuse, alveolar oedema; severe acute, diffuse spleen congestion	lung: moderate infection in the bronchi; heart: moderate infection in the right atrium; ears both sides: severe infection	lung: +++ α-haemolytic streptococci; + *E. coli*; pulmonal lymph node: ++ α-haemolytic streptococci; + *E. coli*; spleen: + α-haemolytic streptococci, *Enterococcus* sp.; liver: ++ coagulase-negative staphylococci; + *E. coli*, *Yarowia lipolytica*; kidney: + *Streptococcus marimammalium*, *E. coli*, β-haemolytic streptococci; intestine: + *Yarowia lipolytica*; mesenteric lymph node: ++ *Yarowia lipolytica*; brain: ++ *Aeromonas* sp.; + *Yarowia lipolytica*, α-haemolytic streptococci; swab of the aorta: +++ *Pseudomonas* spp., *Rahnella aquatilis*; + *Serratia* spp., yeasts; swab of the lung: +++ *Pseudomonas* spp.; swab of the blow hole: +++ *Serratia liquefaciens*, *Pseudomonas* spp., *Ewingella americana*, *Rahnella aquatilis*, *Hafnia alvei*, *Aeromonas* spp., *Shewanella* spp., α-haemolytic streptococci; + yeasts; (+) *Vibrio anguillarum*
92	Pv	yes	16.06.2017	no	mild emaciation; moderate, diffuse, catarrhalic-haemorrhagic enteritis; moderate diffuse catarrhalic-haemorrhagic gastritis; moderate disseminate, catarrhalic-purulent bronchopneumonia; mild, follicular hyperplasia of the spleen; moderate, disseminate, non-purulent hepatitis; mild lymphadenitis simplex of the lymphnodes of the pelvis; moderate hyperaemia of the blood vessels of the brain and the kidneys; moderate congestion of the lung and the liver; mild congestion of the spleen	none	lung: ++ *Carnobacterium maltaromaticum*, a-haemolytic streptococci, *E. coli*; + non-fermenter, *Pantoea agglomerans*, *Lelliottia* species, yeasts, *Mucor* spp.; lung lymphnode: + *Carnobacterium maltaromaticum, Acinetobacter* sp., a-haemolytic streptococci, *E. coli*; spleen: ++ *Carnobacterium maltaromaticum*; + *Bacillus* sp., *Pantoea agglomerans*, yeasts, *Myroides* sp., *E. coli*; liver: ++ *Carnobacterium maltaromaticum*, *Acinetobacter* sp., yeasts; + *E. coli*, *Pantoea agglomerans*, *Proteus* sp.; kidney: ++ *Carnobacterium maltaromaticum*, *Acinetobacter* sp.; +yeasts, *Enterococcus faecalis*, *Pantoea agglomerans*; intestine: + yeasts; mesenteric lymph node: + yeasts, *E. coli*; brain: + *Carnobacterium maltaromaticum*, *E. coli*

93	Pp	no	24.08.2017	yes	emphysema within the abdominal musculature, the pleura, the peritoneum and several organs; haematoma in the blubber and the musculature of the left body wall; haematoma in the area of the left kidney; diarrhoea	Lung: mild infection in the blood vessels; heart: mild infection in the right ventricle; right ear: mild infection	lung: ++ *Proteus* sp., α-haemolytic streptococci, *E. coli*; + *Serratia* sp., *Acinetobacter* sp., *Enterococcus* sp.; spleen: + α-haemolytic streptococci, *E. coli*, *Enterococcus* sp.; intestine: + yeasts; kidney: ++ *Saprolegnia* sp.; + *E. coli*, *Proteus* sp., α-haemolytic streptococci, non-fermenter; liver: + *Saprolegnia* sp., *Proteus* sp., *E. coli*, α-haemolytic streptococci, *Enterococcus* sp.
130	Pp	no	13.07.2017	yes	severely changed, multi-cystic adrenal glands; severe chronic, multifocal cholangohepatitis; small tumour at the diaphragm; several, small skin cuts; mild, subacute, diffuse emphysema of the lung; severe, acute, diffuse, alveolar and interstitial oedema of the lung	lung: moderate infection in bronchi and mild infection in blood vessels; liver: severe infection; ears not determined	lung: ++ *Enterococcus faecalis*; + α-haemolytic streptococci, *E. coli*, *Pseudomonas putida*, *Streptococcus dysgalactiae*, yeasts, *Bacillus* sp.; pulmonal lymph node: ++ *Enterococcus faecalis*; + *Streptococcus dysgalactiae*, *E. coli*, non-fermenter, yeasts, *Proteus* sp.; spleen: ++ *Enterococcus faecalis*; + *Pseudomonas putida*, *E. coli*, yeasts, *Streptococcus dysgalactiae*; liver: ++ *Enterococcus faecalis*; + α-haemolytic streptococci, *E. coli*, *Pseudomonas putida*, yeasts; kidney: ++ *Enterococcus faecalis*; + *Streptococcus dysgalactiae*, α-haemolytic streptococci, *E. coli*, *Bacillus* sp., yeasts, *Pseudomonas putida*, *Proteus* sp.; intestine: -; mesenteric lymph node: yeasts; brain: + *Enterococcus faecalis*, α-haemolytic streptococci, *Pseudomonas putida*, *E. coli*

131	Pp	no	07.08.2017	no	moderate emaciation; netmarks; skin lesions on snout, flipper and fluke; haemorrhages in the blubber; moderate, subacute, multifocal, granulomatous bronchopneumonia; moderate, acute, diffuse emphysema of the lung; haemorrhages in the mucosa of the stomach; mild, chronic, oligofocal, fibrotic cholangitis; severe, acute, diffuse, alveolar lung oedema; mild congestion of the lung	lung: severe infection in bronchi and moderate infection in blood vessels; heart: moderate infection right ventricle; liver: mild infection; ears: bilateral severe infection	lung: ++ *Chryseobacterium indologenes*; + *E. coli*, yeasts, *Klebsiella oxytoca*, *Pseudomonas aeruginosa*; pulmonal lymph node: ++ *Chryseobacterium indologenes*; + *Pantoea* sp., yeasts, *E. coli*, *Pseudomonas aeruginosa*; spleen: + *E. coli*, α-haemolytic streptococci; liver: + *Chryseobacterium indologenes*, *Klebsiella oxytoca*, *E. coli*, *Pseudomonas aeruginosa*; kidney: ++ *Chryseobacterium indologenes*; + *E. coli*, *Serratia marcescens*, yeasts, *Pseudomonas aeruginosa*; intestine: -; mesenterial lymph node: + *E. coli*, yeasts; uterus: + *Chryseobacterium indologenes*, *E. coli*, yeasts, *Pseudomonas aeruginosa*; brain: + *Stenotrophomonas maltophilia*, *Chryseobacterium indologenes*, *E. coli*, *Pseudomonas aeruginosa*
132	Pp	no	29.08.2017	no	linear skin wounds; mild, multifocal, petechial haemorrhages in the subcutis on the ventral abdominal wall; severe, diffuse congestion of the spleen and mesenterium; focal haematoma in the pancreas; mild liver congestion; diffuse haematoma under the capsule of the kidney; moderate congestion of the vessels of the right testis; moderate congestion of the lung; severe, diffuse, alveolar lung oedema; moderate congestion of the vessels of the brain and the meninges; suspicion of by-catch	none	pulomal lymph node: + *Pseudomonas nitroreductans*, *Pseudomonas putida*, *Pantoea agglomerans*, yeasts, *Bacillus* sp., *Enterococcus faecalis*, *E. coli*; lung: + *Stenophomonas maltophilia*, *Pseudomonas putida*, *Serratia* sp., yeasts, *Pantoea agglomerans*, *E. coli*, *Enterococcus faecalis*; brain: + *Pseudomonas nitroreductans*, *Pseudomonas putida*, *Serratia* sp., yeasts, *Enterococcus faecalis*; mesenteric lymph node: + yeasts; intestine: + yeasts; spleen: + *Pseudomonas nitroreductans*, yeasts, *E. coli*, *Enterococcus faecalis*; kidney: + *Pseudomonas nitroreductans*, *Stenotrophomonas maltophilia*, yeasts, *Bacillus* sp., *Proteus* sp., *E. coli*, *Enterococcus faecalis*; liver: + *Pseudomonas nitroreductans*, yeasts, *Pseudomonas putida*, *Lelliottia amnigena*, *E. coli*, *Enterococcus faecalis*

135	Pp	no	04.11.2017	no	severe, acute, diffuse, alveolar bronchopneumonia; moderate, chronic, multifocal, granulomatous bronchopneumonia; mild, chronic, multifocal, necrotizing steatitis and dermatitis; mild, chronic, diffuse, follicular hyperplasia of the white pulpa; mild, chronic, focal, ulcerative gastritis; moderate, chronic, diffuse, necrotizing lymphadenitis of the mesenterial lymph node; severe, acute, diffuse, alveolar oedema of the lung; mild, acute, diffuse, congestion of the liver	lung: severe infection in bronchi and blood vessels; heart: mild infection in the right ventricle; ears: bilateral severe infection	lung: ++ *Vagococcus fluvialis*, *Bacillus* sp., *Pseudomonas putida*; + *E, coli*, coagulase-negative staphylococci, *Enterococcus* sp., *Proteus* sp.; pulmonal lymph node: ++ *E. coli*, *Vagococcus fluvialis*, *Bacillus* sp., *Pseudomonas putida*; + *Enterococcus* sp., *Proteus* sp.; spleen: + α-haemolytic streptococci, *Bacillus* sp., *E. coli*, *Enterococcus* sp.; liver: ++ *Vagococcus fluvialis*, *Bacillus* sp.; + *E. coli*, *Enterococcus* sp.; kidney: + *Bacillus* sp., *Vagococcus fluvialis*, *E. coli*, *Enterococcus* sp.; intestine: + *E. coli*; mesenteric lymph node: -; brain: + *E. coli*, coagulase-negative staphylococci; uterus: + Vagococcus fluvialis, *Serratia marcescens*, *E. coli*, *Enterococcus* sp.

136	Pp	no	16.11.2017	no	severe, acute, diffuse, alveolar bronchopneumonia; severe, chronic, multifocal, granulomatous bronchopneumonia; mild, chronic, oligofocal, granulomatous gastritis (2. compartment); mild, chronic, focal, pyogranulomatous gastritis (4. compartment); severe, chronic, diffuse, granulomatous gastritis (4. compartment); severe, chronic, diffuse, follicular hyperplasia of the white spleen pulpa; severe, chronic, diffuse, necrotizing lymphadenitis of the mesenterial lymph node; mild, chronic, diffuse, cortical hyperplasia of the mesenterial lymph node; severe, acute, diffuse, alveolar oedema of the lung; mild, acute, diffuse congestion of the liver	lung: severe infection in bronchi and moderate infection in the blood vessels; stomach: mild infection; ears: bilateral severe infection	lung: + *Bacillus* sp.; pulmonal lymph node: + coagulase-negative staphylococci; intestine: -; mesenterial lymph node: -; kidney: -; spleen: -; liver: -; brain: -; testis: -

148	Ph	yes	11.06.2017	yes	severe, multifocal, subacute, abscess-forming lymphadenitis; severe, subacute, diffuse bronchopneumonia; severe hyperplasia of the spleen with mild follicular hyperplasia; moderate hypertrophia of the right heart; mild, acute, diffuse, alveolar oedema of the lung	lung: mild nematodes in bronchi; small intestine: mild acanthocephalan	lung: +++ *Hafnia alvei*, *Carnobacterium maltaromaticum*; ++ *Raoultella planticola*, *Enterococcus casseliflavus*, *Streptococcus dysgalactiae*; + *Proteus* sp.; lymphnode of the lung: *Hafnia alvei*, *Carnobacterium maltaromaticum*, *Enterococcus casseliflavus*; ++ *Raoultella planticola*; spleen: +++ *Hafnia alvei*; ++ *Raoultella planticola*, *Enterococcus casseliflavus*; + *Proteus* sp.; liver: *Hafnia alvei*, *Enterococcus durans*; ++ *Raoultella planticola*, anhaemolytic streptococci; + *Proteus* sp.; brain: *Hafnia alvei*, anhaemolytic streptococci, *Carnobacterium maltaromaticum*; ++ *Raoultella planticola*, *Streptococcus dysgalactiae*; + *Proteus* sp.; swab of the abscess: +++ *Hafnia alvei*; + *Enterococcus* sp., a-haemolytic streptococci; abscess: ++ *Hafnia alvei*, *Enterococcus casseliflavus*; + *Raoultella planticola*, *Streptococcus dysgalactiae*, *Clostridium bifermentans*, *Fusobacterium varium*; intestine: ++ *Yarowia lipolytica*, *E. coli*; lymphnode of the intestine: + *E. coli*; kidney: +++ *Hafnia alvei*; ++ *Enterobacter cloacae*, *Streptococcus dysgalactiae*, a-haemolytic streptococci, *Enterococcus* sp.
169	Hg	no	02.07.2018	yes	mild, acute, diffuse, catarrhalic bronchopneumonia; mild chronic, focal, ulcerative colitis; mild, chronic, focal follicular hyperplasia of the mesenteric lymphnode; severe, chronic diffuse hyperplasia of the peyer patches in the ileum; mild, acute, focal, alveolar oedema	small intestine: mild acanthocephalan; colon: severe acanthocephalan	lung: +++ *Psychrobacter sanguinis*; ++ *Streptococcus dysgalactiae*; + *Myroides* sp.; liver: ++ β-haemolytic streptococci, *Psychrobacter sanguinis*, *Myroides* sp.; + *E. coli*, *Enterococcus* sp.; kidney: ++ *Psychrobacter sanguinis*; + *E. coli*, β-haemolytic streptococci; intestine: -; spleen: ++ β-haemolytic streptococci; + *Psychrobacter sanguinis*, *Pseudomonas putida*, anhaemolytic streptococci, *Bacillus* sp.; testis: + β-haemolytic streptococci, *Psychrobacter sanguinis*, *Enterococcus* sp.; brain: + *Streptococcus dysgalactiae*, α-haemolytic streptococci

172	Pp	yes	24.05.2018	no	moderate, chronic, multifocal, granulomatous bronchopneumonia; severe follicular hyperplasia of the spleen; severe, chronic, diffuse, follicular hyperplasia of the mesenterial lymph node; severe, chronic, multifocal bile duct hyperplasia/cholangitis; severe, chronic, diffuse, cortex hyperplasia of the adrenal glands; severe, chronic, diffuse hyperplasia of the thyroid; severe, acute, diffuse, alveolar oedema of the lung	lung: severe infection in bronchi and mild infection in blood vessels; liver: mild infection; ears: bilateral severe infection	lung: +++ *Streptococcus dysgalactiae*; ++ *E. coli*; pulmonal lymph node: +++ *Streptococcus dysgalactiae*; + *E. coli*; spleen: +++ *Streptococcus dysgalactiae*; + *E. coli*; liver: + *Streptococcus dysgalactiae*, *E. coli*; kidney: ++ *Streptococcus dysgalactiae*; + *E. coli*; intestine: + *E. coli*; mesenterial lymph node: + *E. coli*; uterus: +++ *Streptococcus dysgalactiae*; + *E. coli*; mamma: +++ *Streptococcus dysgalactiae; + E. coli*; brain: ++ *Streptococcus dysgalactiae*, *E. coli*; + *Pseudomonas aeruginosa*
193	Pp	yes	02.08.2018	no	mild, acute congestion of the liver; moderate, focal, activation of the lymphatic tissue; severe, acute, diffuse, alveolar oedema of the lung; suspicion of by-catch	none	lung: + *Lelliottia amnigena*, *Klebsiella pneumoniae*, *Proteus* sp.; pulonal lymph node: + *Klebsiella pneumoniae*; spleen: -; liver: -; kidney: + *Lelliottia amnigena*, *Pantoea agglomerans*, *Pseudomonas aeruginosa*, *E. coli*; intestine: -; mesenterial lymph node: -; testis: -
208	Ph	yes	02.09.2018	no	mild, chronic, multifocal, cortical follicular hyperplasia of the mesenteric lymphnode; mild, acute, catarrhalic gastritis of the glandular fundus region; mild, chronic, diffuse, catarrhalic bronchopneumonia; haemoperitoneum; severe, acute, diffuse, alveolar lung oedema; severe, acute, diffuse lung congestion; suspicion of sepsis	small intestine: moderate acanthocephalan	intestine: + *E. coli*; lung: ++ *Acinetobacter* sp.; + *Shewanella putrefaciens*, α-haemolytic streptococci, yeast, *Proteus* sp., *E. coli*; liver: ++ *Lactococcus garvieae*; + *Proteus* sp., *Aeromonas veronii*, *E. coli*; kidney: + *Lactococcus garvieae*, *Morganella morganii*, *Proteus* sp., *E. coli*; spleen: + *Lactococcus garvieae*, α-haemolytic streptococci, *Proteus* sp., *E. coli*; pulmonal lymphnode: ++ *Lactococcus garvieae*; + *Morganella morganii*, *Shewanella putrefaciens*, α-haemolytic streptococci, yeast, *Proteus* sp., *E. coli*; testis: + *Aeromonas* sp., α-haemolytic streptococci, *E. coli*; brain: *Proteus* sp., *Shewanella putrefaciens*, *Lactococcus garvieae*, *E. coli*; mesenteric lymphnode:

210	Ph	no	12.06.2018	no	no necropsy report	stomach/small intestine: tapeworm (unknown severity); colon: acanthocephalan (unknown severity); investigation of other organs unknown	nose: +++ *Shewanella* spp., *Pseudomonas* spp., *Aeromonas* spp., + *E. coli*; vagina: +++ *Pseudomonas* spp., + *E. coli var. haem*., a-haemolytic streptococci
211	Ph	yes	13.06.2018	no	no necropsy report	colon: mild acanthocephalan infection; investigation of other organs unknown	rectum: +++ *Pseudomonas* spp., + *E. coli*, *Shewanella* spp.
212	Ph	yes	14.06.2018	no	no necropsy report	small intestine: acanthocephalan infection of unknown severity; investigation of other organs unknown	rectum: +++ *Pseudomonas* spp., *Shewanella* spp.; ++ *E. coli var. haem*., + *E. coli*, (+) *Clostridium perfringens*
214	Ph	yes	14.06.2018	no	right adrenal glands enlarged + mild hyperplasia of the cortex; cyst in the uterus; multiparus; 5 cm sized cyst left side of the third cervicale vertebrate	small intestine and caecum: mild acanthocephalan infection; negative: lung, heart, liver; unknown: stomach, skin	rectum: +++ *E. coli*, *Pseudomonas* spp., *Clostridium perfringens*; ++*Shewanella* spp.; vagina: +++ Pasteurellaceae, *Pseudomonas* spp.; ++ *E. coli*; + *Shewanella* spp.; uterus: +++ *E. coli*, *Pseudomonas* spp.; left cornus uteri: +++ *E. coli*, *Pseudomonas* spp., *Shewanella* spp.

215	Pv	yes	18.10.2018	yes	cachexia; severe, acute, diffuse, catarrhalic bronchopneumonia; mild, acute, diffuse, interstitial lung emphysema; severe, acute, diffuse, catarrhalic conjunctivitis; mild, acute, diffuse, alveolar lung oedema	heart: mild in the right atrium; small intestine: mild acanthocephalan	lung: +++ *Enterococcus faecalis*; ++ *E. coli*; + *Proteus* sp., yeast, *Myroides* sp.; pulmonal lymphnode: ++ *Enterococcus faecalis*, *E. coli*; + *Proteus* sp., *Myroides* sp.; spleen: ++ *Enterococcus faecalis*, *E. coli*; + *Proteus* sp., *Myroides* sp.; liver: +++ *Enterococcus faecalis*; ++ *E. coli*; + Proteus sp., *Myroides* sp.; kidney: +++ *Enterococcus faecalis*; ++ *E. coli*; + *Proteus* sp., *Myroides* sp., yeast; intestine: ++ *E. coli*; + yeast; swab of the eye: +++ *Pseudomonas* sp., *E. coli*, *Pantoea agglomerans*; ++ *Enterococcus faecalis*, *Chryseobacterium scophthalmum*; + *Proteus* sp., *Myroides* sp.; uterus: +++ *Enterococcus faecalis*; ++ *E. coli*, yeast; + *Proteus* sp., *Myroides* sp.; brain: +++ *Enterococcus faecalis*; ++ *Raoultella planticola*, *E. coli*; + *Proteus* sp., *Myroides* sp.
216	Pp	no	29.10.2018	yes	moderate emaciation; luxation of 3 ribs; haemorrhages in the subcutis and rete mirabile; mild lung congestion; suspicion of trauma	none	not determined
218	Pp	no	15.11.2018	yes	moderate focal haemorrhages in the acoustic fatty tissue of the left side; moderate, oligofocal, areal hypertrophia of the mucosa of the small intestine; mild, oligofocal liver cell necrosis; suspicion of by-catch	none	lung: +++ *Lelliottia amnigena*; ++ *Enterococcus faecalis*; + *E. coli*; pulmonal lymph node: ++ *Lelliottia amnigena*, *Enterococcus faecalis*; + *E. coli*, *Proteus* sp., *Myroides* sp.; spleen: ++ *Enterococcus faecalis*; + *E. coli*, *Pantoea agglomerans*, α-haemolytic streptococci; liver: ++ *Lelliottia amnigena*, *Enterococcus faecalis*; + *E. coli*, *Pantoea agglomerans*; kidney: ++ *Lelliottia amnigena*, *Enterococcus faecalis*; + *E. coli*, *Pantoea agglomerans*; intestine: -; mesenterial lymph node: + *E. coli*; testis: ++ *Lelliottia amnigena*, *Lactococcus garvieae*, *Enterococcus faecalis*; + *Pantoea agglomerans*, *Myroides* sp., *E. coli*

221	Pp	no	17.11.2018	no	severe, chronic, multifocal, granulomatous bronchopneumonia; mild, chronic, focal, pyogranulomatous bronchopneumonia; severe, chronic, diffuse, granulomatous gastritis (4. compartiment); mild chronic, focal, necrotizing steatitis; mild, chronic, focal, pyogranulomatous statitis; severe, acute, diffuse, follicular hyperplasia; severe, acute, diffuse hyperplasia of the tonsil; severe, acute, diffuse, alveolar oedema of the lung; mild, acute, diffuse congestion of the liver; by-catch	lung: severe infection in the bronchi and moderate infection in the blood vessels; heart: mild infection of the right atrium; liver: mild infection; ears: severe infection bilateral	lung: ++ *Acinetobacter* sp.; + *Proteus* sp., *E. coli*, α-haemolytic streptococci, anhaemolytic streptococci, *Streptococcus phocae*, E*nterococcus* sp.; pulmonal lymph node: ++ *Psychrobacter sanguinis*; + *E. coli*, α-haemolytic streptococci, anhaemolytic streptococci, *Streptococcus phocae*, *Pseudomonas rhodesiae*, *Enterococcus* sp., *Proteus* sp.; spleen: ++ *Psychrobacter sanguinis*; + *E. coli*, coagulase-negative streptococci, *Enterococcus faecalis*; liver: + *Psychrobacter sanguinis*; *E. coli*, coagulase-negative streptococci, *Pseudomonas rhodesiae*, *Enterococcus faecalis*, *Proteus* sp.; kidney: ++ *E. coli*, anhaemolytic streptococci; + *Proteus* sp., α-haemolytic streptococci; intestine: -; mesenterial lymph node: -; stomach: ++ *Psychrobacter sanguinis*, *Penicillium* sp.; + *E. coli*, anhaemolytic streptococci, *Proteus* sp., *Enterococcus faecalis*, *Yarowia lipolytica*; testis: + *Enterococcus faecalis*, *Enterococcus faecium*, *Pantoea agglomerans*; brain: + *Staphylococcus lugdunensis*, *Pseudomonas putida*

222	Pp	yes	03.12.2018	no	severe, chronic, diffuse, granulomatous bronchopneumonia; cholangitis, pericholangitis and proliferation of the bile ducts; mild, acute, diffuse, ulceration of the oesophagus; severe, chronic, diffuse, granulomatous lymphadenitis of the mesenterial lymph node; suspicion of acute, diffuse cystitis; suspicion of acute, diffuse tonsilitis bilateral; suspicion of mild, acute, multifocal plaques of the stomach mucosa; multiple net marks; severe, acute, diffuse, alveolar, oedema of the lung; severe, acute, diffuse congestion of the lung and liver; by-catch	lung: severe infection in the bronchi and moderate infection in the blood vessels; ears: severe infection bilateral	lung: + *Enterococcus* sp., Morganella morganii; pulmonal lymph node: + *Enterococcus* sp.; spleen: -; liver: -; kidney: + coagulase-negative staphylococci, *Pseudomonas* sp., *Enterococcus* sp.; intestine: -; mesenterial lymph node: -; testis: + non-fermenter; brain: + *Enterococcus* sp.

223	Pv	yes	06.12.2018	yes	cachexia; moderate, subacute, diffuse, granulomatous bronchopneumonia; wounds on the head; diarrhoea; mild cystitis; mild, acute, diffuse lung oedema	lung: moderate infection in bronchi and blood vessels; small intestine: mild acanthocephalan	lung: +++ *Streptococcus phocae*; + *Brucella* sp., *Proteus* sp., *Pantoea agglomerans*, *E. coli*, *Enterococcus* sp.; pulmonal lymph node: ++ *Streptococcus phocae*, anhaemolytic streptococci; + *Proteus* sp., *Pantoea agglomerans*, *E. coli*, *Enterococcus* sp., moult fungus; spleen: ++ *Streptococcus phocae*; + *Proteus* sp., *Pantoea agglomerans*, *E. coli*, *Enterococcus* sp., moult fungus; liver: ++ *Streptococcus phocae*; + *Proteus* sp., *Pantoea agglomerans*, *E. coli*, *Enterococcus* sp., moult fungus; kidney: ++ *Streptococcus phocae*, *Streptococcus dysgalactiae*, *Pantoea agglomerans*; + *Proteus* sp., *E. coli*, *Enterococcus* sp.; intestine: + *E. coli*; mesenterial lymph node: + *E. coli*; testis: + *Pantoea agglomerans*, *E. coli*, *Enterococcus* sp.; brain: +++ *Streptococcus phocae*; ++ *Pantoea agglomerans*, *Proteus* sp., *E. coli*, *Enterococcus* sp.; + moult fungus
226	Ph	no	04.06.2019	no	moderate endoparasitosis	small intestine: moderate acanthocephalan (in the middle part); large intestine: mild acanthocephalan; part small to large intestine: severe acanthocephalan	liver: (+) *Staph. saprophyticus*, *Athrobacter* spp., *Microcioccus luteus*; spleen: (+) α-haemolytic streptococci; kidney: (+) *Pseudomonas* spp.; Lunge: + *Pseudomonas* spp., α-haemolytic streptococci, *Pasteurellacae*; pulmonal lymphnode: (+) α-haemolytic streptococci; intestine: (+) *Pseudomonas* spp., + *Psychrobacter* spp., *Kocuria* spp., *Clostridium perfringens*; mesenterial lymphnode: (+) *Pseudomonas* spp., + *Psychrobacter* spp.; CNS: (+) *Pseudomonas* spp., (+) *Pseudomonas* spp., + *Psychrobacter* spp.; rectal swab: + *Pseudomonas* spp.

227	Ph	yes	05.06.2019	no	serositis spleen, moderate endoparasitosis	small intestine: moderate acanthocephalan; large intestine: moderate acanthocephalan	liver: (+) *E. coli*; spleen: (+) *E. coli*, *E. coli var. haemolytica*; kidney: (+) *E. coli*; lung: (+) *E. coli*, coryneform bacteria; pulmonal lymphnode: (+) coryneform bacteria; intestine: +++ *Clostridium perfringens*; mesenteric lymphnode: (+) *E. coli*; uterus: + *Sc. dysgalactiae*; rectal swab: +++ *Cl. pefringens*, *Providencia rettgeri*, *Pseudomonas* spp., ++ *E. coli*; vaginal swab: +++ *Pseudomonas* spp., + *E. coli*, *Cl. perfringens*
228	Ph	yes	05.06.2019	no	moderate chronic colitis; severe infection with acanthocephalan; mild, acute, diffuse, granulomatous pneumonia; mild follicular hyperplasia of the spleen; moderate hyperplasia of the adrenal cortex	small intestine: second half with mild acanthocephalan; large intestine: severe acanthocephalan	spleen and lung: (+) α-haemolytic streptococci, coryneform bacteria; testis: (+) coryneform bacteria; rectal swab: +++ *Shewanella* spp., + *E. coli*, *Sc. dysgalactiae*
229	Ph	no	05.06.2019	no	diarrhoea; moderate infection with acanthocephalan; mild, acute, oligofocal, granulomatous pneumonia	small and large intestine: moderate acanthocephalan	spleen: *Staph. warneri*, *Sc. dysgalactiae* ssp. *equisimilis*, α-haemolytic streptococci; kidney and intestine: (+) *Sc. dysgalactiae* ssp. *equisimilis*; testis: + *Sc. dysgalactiae* ssp. *equisimilis*; rectal swab: +++ *Pseudomonas* spp.
230	Ph	no	06.06.2019	no	moderate, chronic, ulcerative colitis; moderate to severe infection of acanthocephalan; ovarian cyst (left ovary)	small intestine: severe acanthocephalan; large intestine: moderate acanthocephalan	liver, spleen, kidney, mesenterial lymphnode: + *E. coli*, *E. coli var. haem.*; lung, pulmonal lymphnode, CNS, uterus: (+) *E. coli*, *E. coli var. haem.*; intestine: (+) *E. coli var. haem.*; rectal swab: +++ *E. coli*, *Cl. perfringens*, *Acinetobacter* spp.; + *E. coli var. haem.*; vaginal swab: +++ *E. coli*, *Stenothrophomonas* spp.; + *E. coli var. haem.*, *Pantoea* spp.
231	Ph	no	07.06.2019	no	moderate infection with acanthocephalan; mild, acute, diffuse pneumonia; pulmonal lymphadenitis; moderate follicular hyperplasia of the spleen; abrasion of the claws of the forelimbs; mild oesophagitis	small intestine and colon: moderate acanthocephalan	intestine: (+) *Cl. perfringens*; rectal swab: ++ *Cl. perfringens*, + *Pseudomonas* spp., *Corynebacterium* spp., α-haemolytic streptococci; vaginal swab: no growth

232	Ph	yes	07.06.2019	no	ovarian cyst (left ovary); moderate, chronic enteritis; mild, diffuse, catarrhalic enteritis; mild acanthocephalan infection; mild follicular hyperplasia of the spleen; mild lung emphysema	small intestine: mild acanthocephalan; large intestine: mild acanthocephalan	spleen, kidney: (+) *E. coli*, *E. coli var. haemolytica*; intestine: (+) *E. coli*, *E. coli var. haemolytica*, *Cl. perfringens*; lung: + *Corynebacterium* spp., (+) α-haemolytic streptococci, *Bisgaardia hudsonensis*; mesenteric lymphnode: (+) *E. coli*; Uterus: (+) *Pseudomonas* spp., *Moraxella* spp.; rectal swab: ++ *E. coli*, *Cl. perfringens*, + *E. coli var. haem.*; vaginal swab: +++ *Pseudomonas* spp., + *E. coli*, *E. coli var. haem.*
233	Ph	yes	07.06.2019	no	moderate infection with acanthocephalan; mild emphysema of the lung; mild follicular hyperplasia of the spleen	small intestine: moderate acanthocephalan; large intestine: mild acanthocephalan	kidney, lung, pulmonal lymphnode: (+) *Pseudomonas* spp.; liver: (+) *E. coli*; spleen: (+) *Staph. equorum*; intestine: + *Cl. perfringens*, (+) *Pseudomonas* spp., aerobic *bacillus* spp.; uterus: + *E. coli var. haem.*, (+) *Pseudomonas* spp., α-haemolytic streptococci; rectal swab: +++ *Hafnia alvei*, *E. coli var. haem.*, (+) *Cl. perfringens*; vaginal swab: +++ *Hafnia alvei*, *Pseudomonas* spp., ++ yeasts, + *Pantoea* spp., *Cl. perfringens*
234	Ph	yes	07.06.2019	no	moderate body condition; moderate infection with acanthocephalan; moderate, chronic cholecystitis; moderate fibrosis of the left atrioventricular valve	small intestine: mild acanthocephalan; colon: moderate acanthocephalan	lung: (+) Pasteurellaceae; CNS: (+) α-haemolytic streptococci, *Pseudomonas* spp.; *Psychrobacter* spp.; rectal swab +++ *E. coli var. haem.*, *Pseudomonas* spp., (+) *Aeromonas* spp.
235	Ph	no	08.06.2019	no	moderate infection with acanthocephalan; mild, focal atelectasis of the lung	small intestine: mild acanthocephalan; colon: moderate acanthocephalan	lung: + *Neisseria* sp., (+) *Sc. dysgalactiae* spp. *equisimilis*; pulmonal lymphnode: (+) Neisseria sp., *E. coli var. haem.*; intestine: *Cl. perfringens*; CNS: + *Neisseria* spp., *Corynebacterium* spp., *E. coli. var. haem.*; uterus: (+) *E. coli var. haem.*; rectal swab: +++ *Pantoea* spp., *Shewanella* spp., *Pseudomonas* spp., (+) yeasts; vaginal swab: + *Staphylococcus* spp

Table S3: Necropsy findings. The table lists all necropsied marine mammals, their sample IDs, the species (Pv = *Phoca vitulina* (harbour seal), Hg = *Halichoerus grypus* (grey seal), Ph = *Phusa hispida*, Pp = *Phocoena phocoena* (harbour porpoise)), if antimicrobial-resistant *E. coli* were isolated from the sample, date of necropsy, freezing prior to necropsy, main macroscopic findings, parasitic burdens, and results of microbiology testing of tissues.
