# Supplementary material for: Occurrence of Antimicrobial-Resistant Escherichia coli in Marine Mammals of the North and Baltic Seas: Sentinels for Human Health
Source: Antibiotics (Basel). 2022 Sep 14;11(9):1248. doi: 10.3390/antibiotics11091248 (PMC9495373; doi:10.3390/antibiotics11091248)
Supplement: Supplementary file 1 [file antibiotics-11-01248-s001.zip › Table S4.pdf]

ID	Resistance	Histopath lung	Histopath liver	Histopath spleen	Histopath kidney	Histopath intestine	Histopath others
1	no	mild intraalveolar and intrabronchial nematode infection; suspicion on pneumonia (focal mild, basophilic hypercellular area); lobular restricted, subtotal atelectasis; moderate, alveolar oedema; mild, alveolar emphysema; CAVE: advanced autolyses	not determined	not determined	not determined	not determined	brain: focal, severe haemorrhages (caused by the shot)
56	yes	multifocal, moderate, alveolar and interstitial oedema	within normal limits	within normal limits	within normal limits	within normal limits	stomach: moderate to severe, mural gastritis (poor decomposition status); mesenterial lymph node: moderate, focal, eosinophilic infiltrates; bone marrow: moderate cell rich, all 3 cell rows present
79	no	multifocal, severe, proof of intralobular and intrabronchial parasites (adult and larvae of nematodes); lobular restricted, subtotal atelectasis; multifocal, mild, alveolar oedema and emphysema	within normal limits	within normal limits	within normal limits	lamina propria with mild multifocal, eosinophilic inflammation with associated intramucosal parasites	lung lymph node: multifocal, basophilic, hypercellular areas with intralesional parasites (suspicion of lymphadenitis; autolysis); abdominal wall: oligofocal, mild, nests forming, lymphocytic infiltrates; multifocal mild, hyalinic degeneration; within normal limits: adrenal glands, diaphragm, pancreas, stomach, mesenterial lymph node

80	yes	multifocal, severe proof of intralobular and intrabronchial parasites (adult and larvae of nematodes) with associated, multifocal, hypercellular areas (suspicion of pneumonia; autolysis); lobular restricted, subtotal atelectasis; mild alveolar emphysema	within normal limits	within normal limits	within normal limits	large intestine: lamina propria with multifocal mild infiltrates of eosinophilic granulocytes	stomach: focal, severe, extensive, ulcerative gastritis with intralesional parasites
81	no	multifocal, moderate to severe intralobular and intrabronchial parasites (adults and larvae of nematodes) with lobular restricted, subtotal atelectasis; multifocal, moderate, alveolar emphysema; moderate, multifocal, alveolar oedema	within normal limits	within normal limits	not determined	not determined	pulmonal lymphnode: within normal limits
82	yes	multifocal, mild, alveolar oedema and emphysema	within normal limits	within normal limits	within normal limits	intraluminar proof of single nematodes (autolysis)	none
83	no	severe, diffuse, alveolar and interstitial oedema; multifocal mild to moderate, alveolar emphysema	within normal limits (autolysis)	within normal limits (autolysis)	not determined	small intestine within normal limits (autolysis)	bone marrow: moderate cell rich (autolysis); skin: segmental, severe compression; stomach: suspicion of ulcerative and granulomatous gastritis with intralesional foreign material (autolysis); within normal limits: sclera, heart, lung lymph node (autolysis), musculature

85	no	severe, alveolar oedema; mild alveolar emphysema; mild aspirated keratin lamellae; mild to moderate alveolar histiocytosis	within normal limits (autolysis), no vacuolisation of hepatocytes (no fat depots)	not determined	not determined	not determined	brain: cerebellum with outer grain cell layer, cerebrum within normal limits; within normal limits: eye, musculature, skin, rete mirabile
86	yes	diffuse, mild, alveolar, oedema and emphysema; oligofocal moderate to severe, alveolar focal pneumonia (circumsciptal hypercellularity, autolysis)	within normal limits (autolysis)	white pulpa cell rich; within normal limits	within normal limits	within normal limits	skin: focal mild to moderate, epidermal hyperplasia with loss of epidermal pigmentation and mild pigment incontinence; within normal limits: spinal cord (artefacts), eye, adrenal gland, pulmonal and mesenterial lymph node (autolysis), retropharyngeal lymph node, musculature, diaphragm, urinary bladder, brain, tongue, cutaneous mucosa, heart, trachea, rete mirabile, aorta stomach, pancreas (autolysis)

92	yes	mild alveolar histiocytosis	mild, diffuse vacuolation of hepatocytes; multifocal, mild pigment storage in hepatocytes and macrophages; mild dissociation of liver cell bars; multifocal, mild, single cell necrosis	white pulpa moderate cell rich; moderate, multifocal extramedullar haematopoiesis	mild to moderate hyperaemia	no result	skin: moderate, extravascular erythrocytes on the skin surface and in superficial hair follicles; pulmonal and mesenteric lymphnode: multifocal, mild, acute haemorrhage; oesophagus: severe, focal, acute haemorrhages in the surrounding tissue; trachea: acute haemorrhages in the surrounding tissue, few amount of extravasal erythrocytes in the lumen; brain: cerebrum with multifocal, mild, partly perivascular, acute haemorrhages, few vacuoles in the neurons; spinal cord: multifocal, mild, acute haemorrhages; adrenal glands: moderate, multifocal, acute haemorrhages in the surrounding tissue, mild hyperaemia; within normal limits: pancreas, skeletal muscles, tongue, retropharyngeal lymphnode, tonsils, aorta, heart, cornea, sclera, uvea, retina, thyroid, thymus
93	no	mild intravascular nematodes without perifocal, reactive changes; severe, diffuse alveolar oedema	suspicion of oligofocal non-reactive caverna (suspicion of gas bubbles; autolysis)	within normal limits	within normal limits	moderate, diffuse hypercellularity of the lamina propria (cytological details not identifiable; autolysis)	musculature with focal moderate fibrosis; skin with multiple, connective tissue demarcating cavernas in the deeper layer of the blubber

130	no	multifocal mild, lymphocytic-plasmacellular bronchitis; focal moderate, chronic, granulomatous and eosinophilic pneumonia; focal moderate media hypertrophia of one artery	severe, chronic, lymphocytic-plasmacellular and eosinophilic, fibrotic cholangitis with intralesional trematode eggs	not determined	multifocal mild concrements in the distal canaliculi; multifocal segmental or total sclerotizing glomerulonephritis with atrophy of glomerular canaliculi; moderate PAS-positive protein cylinder in distal canaliculi	within normal limits (autolysis)	adrenal glands bilateral with multifocal, subcortical cysts; pulmonal lymph node with mild anthracosis; thymus with multiple cysts (thymus not sampled in this animal); within normal limits: heart, pancreas, mesenterial lymph node (autolysis)
131	no	focal severe, chronic-active, purulent bronchopneumonia with granulomatous parts and multifocal, severe, follical-like aggregated, mainly lymphocytic, interstitial infiltrates; multifocal, squamous metaplasia of the bronchi epithelia; multifocal, severe, chronic, granulomatous and eosinophilic pneumonia with intralesional nematodes; moderate, alveolar oedema; multifocal, amphiphilic deposits in the area of the bronchial epithelium	multifocal, mild, granulo-histiocytic and lymphocytic, portal hepatitis; multifocal mild proliferation of the bile duct; multifocal moderate to severe, chronic, proliferative, lymphocytic-plasmacellular and eosinophilic cholangitis and pericholangitis with intralesional trematodes and trematodes eggs	moderate, follicular hyperplasia	within normal limits	within normal limits	tonsil: severe, follicular hyperplasia; stomach: pars proventricularis within normal limits, pars glandularis with mild to moderate, lymphocytic-plasmacellular gastritis, multifocal moderate, follicular, mainly lymphocytic gastritis; eye: focal at the limbus mild, fresh haemorrhages, other parts within normal limits; mesenterial lymph node with severe, follicular hyperplasia; spinal cord with mild fresh bleeding; within normal limits: brain, tongue, adrenal gland, thymus (cell rich), aorta, rete mirabile, musculature, diaphragm, pulmonal lymph node, trachea, pancreas, thyroid, oesophagus, heart, bone marrow, urinary bladder

132	no	diffuse, mild, alveolar histiocytosis	diffuse mild, centrolobular steatosis	mild hyperaemia	moderate, interstitial haemorrhages, perirenal severe haematoma	within normal limits	mesenterial lymph node and tonsil: moderate, follicular hyperplasia; adrenal gland: severe, perirenal haematoma; skin: oligofocal complete defect of the epidermis with corresponding haemorrhages in the subepidermal connective tissue; within normal limits: brain, pulmonal and retropharyngeal lymph node, tongue, thyroid, thymus, trachea, oesophagus, spinal cord, aorta, rete mirabile, heart, diaphragm, pancreas, stomach, urinary bladder, blubber, musculature eye

135	no	severe, pulmonal, endoparasitosis with nematodes in blood vessels, airways and parenchyma; multifocal moderate hypertrophia and fibrosis of blood vessel walls; multifocal, moderate, chronic, granulomatous and eosinophilic pneumonia with intralesional nematodes; focal thrombosis in organisation; focal severe, chronic, granulomatous pneumonia with fibrosis and central dystrophic calcification; multifocal moderate, eosinophilic to amphophilic, irregular deposits in the bronchial epithelium; single, completely obliterate vessels with severe hyalinosis of the wall; mild to moderate, alveolar oedema	within normal limits	within normal limits	within normal limits	within normal limits	thymus with mild atrophia; stomach pars proventricularis within normal limits, pars glandularis with focal, mainly lymphocytic gastritis; diffuse moderate, lympho-plasmacytic tracheitis with moderate, epithelial hyperplasia; skin (5x): severe, granulomatous and necrotizing panniculitis; focal acute, moderate, ulcerative and purulent dermatitis; focal severe ulceration with chronic granulation tissue, haemorrhages and perifocal, epidermal hyperplasia; focal severe, chronic-active, ulcerative and purulent dermatitis and panniculitis with intralesional foreign material and perifocal fibrosis; focal erosions and ulceration with mild bleeding and mild granulomatous leukocytosis; brain with single mild, perivascular, acute haemorrhages; within normal limits: hypophysis, thyroid, eye, spinal cord, heart, adrenal gland, tongue, pancreas, musculature, diaphragm, retropharyngeal and pulmonal lymph node, aorta, urinary bladder

136	no	moderate pulmonal endoparasitosis with nematodes in the parenchyma; multifocal mild to severe, granulomatous and eosinophilic pneumonia with intralesional nematode larvae; multifocal arteria completely filled with collagen fibre-rich conjunctive tissue with central necrosis and dystrophic calcification; focal severe thrombosis and pyogranulomatous arteritis; diffuse to moderate, alveolar oedema; multifocal moderate, hyaline to fine granular, amphophile deposits in the bronchial epithelium; moderate hyperaemia	mild, acute congestive hyperaemia	mild, follicular hyperplasia	within normal limits	moderate, follicular hyperplasia of the Peyer patches; mucosa and intestinal wall within normal limits	pulmonal lymph node with severe, follicular hyperplasia; mesenterial lymph node with moderate, follicular hyperplasia and focal moderate, granulomatous and eosinophilic lymphadenitis; stomach pars proventricularis within normal limits, pars glandularis with multifocal severe, follicular gastritis and multifocal bis comfluent, moderate to severe, lympho-plasmacytic, mural gastritis; tonsil with severe, follicular hyperplasia; skin focal with mild, crater-shaped compression of the epidermis, skin with one linear and one irregular, non-reactive margin, skin with focal complete, non-reactive loss of epidermis; diffuse mild, lympho-plasmacytic and eosinophilic tracheitis; within normal limits: thymus, retropharyngeal lymph node, thyroid, spinal cord, brain, hypophysis, eye, adrenal glands, heart, tongue, oesophagus, urinary bladder; rete mirabile, pancreas, musculature, diaphragm
148	yes	mild, pulmonal endoparasitosis with nematodes in the deep airways; severe alveolar and subpleural oedema; moderate interstitial oedema	within normal limits (autolysis)	within normal limits	within normal limits (autolysis)	small and large intestine: within normal limits (autolysis)	within normal limits: heart and skin

169	no	severe, diffuse, alveolar and interstitial oedema; disseminated, mild, alveolar emphysema	within normal limits (autolysis)	within normal limits (autolysis)	within normal limits (autolysis)	large intestine: within normal limits	mesenterial lymphnode: focal, moderate, eosinophilic lymphadenitis; moderate putrefactive emphysema; suspicion of moderate to severe, diffuse, eosinophilic, capsular and perinodular inflammation
172	yes	severe hyperaemia; severe, alveolar oedema; segmental mild, lympho-histiocytic and plasmacellular pleuritis with severe fibrosis; moderate to severe alveolar histiocytosis with macrophages with ferrous pigment in the cytoplasm especially in the subpleural and peribronchial area; mild hyperplasia of the bronchus-associated, lymphatic tissue; focal severe, chronic, lympho-histiocytic and plasmacellular pneumonia with severe fibrosis	disseminated mild, hepatocellular pigment-storage hepatosis; multifocal mild, chronic, lymphocytic-plasmacellular, interstitial hepatitis with fibrosis and mild bile duct proliferation	mild, extramedullar haematopoiesis	within normal limits	diffuse mild to moderate, lymphocytic-plasmacellular and eosinophilic infiltration of the mucosa	thyroid with individual cysts; stomach: pars proventricularis with moderate, parakeratotic hyperkeratosis, multifocal mild, lymphocytic-plasmacellular gastritis, oligofocal mild to moderate, intracellular oedema in the epithelial cells; pars glandularis with moderate hyperaemia; focal, moderate, lympho-histiocytic and plasmacellular myositis; mesenterial lymph node with mild to moderate fibrosis, suspicion of mild to moderate, lymphocytic depletion; pulmonal lymph node with severe, acute haemorrhages; diffuse moderate to severe, lymphocytic-plasmacellular tracheitis; cutanic mucosa with diffuse, mild to moderate, lymphocytic-plasmacellular infiltration and mild lymphocytic-plasmacellular sialoadenitis (tonsil); within normal limits: adrenal glands, pancreas (autolysis), diaphragm, eye, brain, mamma, skin, heart, rete mirabile, aorta, urinary bladder, spinal cord, bone marrow

193	yes	mild, alveolar oedema	mild, acute congestive hyperaemia; diffuse mild, vacuolisation of hepatocytes (suspicion of fatty liver)	moderate hyperaemia	severe, acute, perirenal haemorrhages; moderate interstitial haemorrhages	Peyer patches cell rich; within normal limits	tonsil moderate, follicular hyperplasia; pulmonal lymph node with moderate, follicular hyperplasia; retropharyngeal lymph node with mild, follicular hyperplasia; within normal limits: hypophysis, mesenterial lymph node, tongue, urinary bladder, adrenal glands, trachea, aorta, bone marrow, heart, musculature, diaphragm, oesophagus, brain, stomach, thymus, rete mirabile, eye, thyroid, skin
208	yes	lung with severe, diffuse haemorrhages	focal, severe haemorrhages in the parenchyma (autolysis)	mild, extramedullar haematopoiesis	within normal limits	small and large intestine: within normal limits	adrenal glands: mild to moderate hyperaemia at the cortico-medullar passage; pulmonal lymphnode: moderate to severe, follicular hyperplasia; mesenterial lymphnode: mild follicular hyperplasia; retropharyngeal lymphnode: moderate, follicular hyperplasia; thymus: cell rich; focal, moderate haemorrhages in the lymphatic tissue; tonsil: severe, follicular hyperplasia; within normal limits: trachea, musculature, diaphragm, brain, heart, urinary bladder, tongue, skin, thyroid, stomach, oesophagus, eye, pancreas, spinal cord (autolysis)
210	no	severe acute lung haemorrhages; focal, moderate, granulomatous pneumonia; oligofocal mild-moderate, lympho-plasmacytic peribonchitis; multifocal moderate, alveolar and interstitial emphysema	multifocal, mild lymphocytic hepatitis	within normal limits	within normal limits	within normal limits	follicular hyperplasia of lymphatic tissues (lymphnodes, tonsils, Peyer plates)

211	yes	lung with severe acute bleeding in the parenchyma + focal moderate, chronic, lobular overarching, granulomatous pneumonia + multifocal mild, perivascular, lympho-plasmacytic, interstitial pneumonia + mild, alveolar oedema	liver with mild hyperaemia	spleen with severe, follicular hyperplasia	kidney with moderate mineralised concrements in the distal tubuli	small intestine with severe follicular hyperplasia of the Peyer plates; stomach with multifocal mild to moderate, partly follicular aggregating, lymphocytic dominated gastritis; tonsils with severe, follicular hyperplasia; lymphnode (location unknown) with moderate to severe, follicular hyperplasia; skin with mild, lympho-histiocytic, perivascular dermatitis superficialis	tongue with oligofocal, mild, chronic-active, lympho-plasmacytic glossitis

212	yes	mild, pulmonal endoparasitosis with nematodes in the parenchyma; moderate, acute, diffuse haemorrhages in the parenchyma; multifocal, mild, alveolar, focal severe, interstitial emphysema	focal moderate, lympho-histiocytic hepatitis	moderate, follicular hyperplasia	within normal limits	small intestine: severe, follicular hyperplasia of the peyer patches; large intestine: within normal limits	sclera with segmental, severe oedema and moderate haemorrhages; tonsil with severe, follicular hyperplasia; multifocal, moderate to severe granulomatous and pyogranulomatous lymphadenitis (lymph node not named) and two lymph nodes with severe, follicular hyperplasia; within normal limits: adrenal gland, brain urinary bladder, thymus, pancreas, stomach, musculature, diaphragm, thyroid, trachea, aorta, heart, tongue, bone marrow, oesophagus, skin

214	yes	severe hyperaemia; focal, severe, alveolar and interstitial, acute haemorrhages; focal, mild, non-reactive, pulmonal endoparasitosis with nematodes in the alveoli	multifocal, mild to moderate, centro-lobular accentuated, eosinophilic degeneration of hepatocytes; mild to moderate deposition of pigment in centro-lobular hepatocytes	mild, extra medullar haematopoiesis	within normal limits	small intestine: mild follicular hyperplasia of the Peyer's patches; large intestine: within normal limits	tonsil: cutaneous mucosa with multifocal mild to moderate, nest-like arranged, lymphocytic cells; skin: multifocal to confluent, mild to moderate, perivascular accentuated, mainly lymphocytic dermatitis with mild to moderate pigment incontinence; multifocal, mild to moderate, periadnexal, mainly lymphocytic dermatitis with evidence of an intrafollicular, parasite-like structure; focal, moderate, lympho-histiocytic gastritis; thymus cell rich with single cysts; pulmonal lymph node with severe, follicular hyperplasia; mesenteric lymph node with mild, follicular hyperplasia and multifocal, mild deposits of black pigment; adrenal glands with severe, nodular, cortical hyperplasia; heart with multifocal, especially in the myocardium of the left ventricle, moderate loss of heart muscle fibres with compensatory collagen fibre containing connective tissue (Azan staining: positive); perifocal cardiomyocytes with extensive karyomegaly; conjunctive with mild to moderate, chronic-active, lympho-histiocytic inflammation; within normal limits: tongue, trachea, brain, musculature, eye, pancreas, thyroid, oesophagus, urinary bladder, aorta

215	yes	moderate, alveolar oedema; mild, interstitial oedema; multifocal, mild, alveolar emphysema	within normal limits (autolysis)	within normal limits (autolysis)	within normal limits (autolysis)	small intestine: focal mild, perivascular hypercellularity (suspicion of perivascular inflammation (autolysis); large intestine: within normal limits	pulmonal lymphnode: within normal limits (autolysis)
216	no	moderate, alveolar oedema; mild, interstitial oedema	within normal limits (autolysis)	within normal limits (autolysis)	within normal limits (autolysis)	within normal limits (autolysis)	within normal limits: skin, adrenal glands (autolysis)
218	no	mild alveolar oedema	within normal limits (autolysis)	within normal limits	within normal limits (autolysis)	prominent peyer patches	skin with non-reactive loss of epidermis; within normal limits: mesenterial and retropharyngeal lymph nodes, stomach, heart, adrenal glands, aorta, rete mirabile, tongue, urinary bladder, thymus, trachea, musculature, spinal cord, eye, pancreas

221	no	moderate, pulmonal endoparasitosis with nematodes in arteries and in the lung parenchyma; multifocal severe, granulomatous and eosinophilic pneumonia with intralesional parasites; multifocal, moderate, alveolar histiocytosis; mild to moderate lympho-plasmacytic, interstitial pneumonia; multifocal severe hyperplasia of the bronchus-associated lymphatic tissue; mils alveolar oedema; multifocal moderate, lympho-plasmocytic bronchitis; severe hyperaemia	multifocal severe, granulomatous cholangitis and pericholangitis with solitary intralesional eggs of trematodes; severe, follicular, mainly lymphocytic pericholangitis	moderate follicular hyperplasia	moderate hyperaemia	diffuse, mild, lympho-plasmacytic and eosinophilic infiltration of the lamina propria	spinal cord in ventral funiculi solitary dilatated myelin sheath with swollen axons; brain in rostral brain stem solitary perivascular, acute, mild, haemorrhages; auditory cortex within normal limits; pulmonal and retropharyngeal lymph node with severe follicular hyperplasia; mesenterial lymph node with moderate follicular hyperplasia, multifocal moderate granulomatous and eosinophilic lymphadenitis, mild eosinophilic lymphadenitis, moderate perinodal and capsular, mainly eosinophilic inflammation; adrenal glands with mild nodular hyperplasia; tonsil with severe follicular hyperplasia; stomach: pars proventricularis within normal limits, pars glandularis with multifocal moderate follicular mainly lymphocytic gastritis, multifocal mild to moderate, lympho-plasmacellular and eosinophilic gastritis; skin with multifocal mild to moderate, acute haemorrhages in the blubber; within normal limits: musculature, diaphragm, rete mirabile, thymus, aorta, tongue, oesophagus, pancreas, hypophysis, urinary bladder, trachea, heart, eye, bone marrow

222	yes	mild, pulmonal endoparasitosis with nematodes in the deeper airways and vessels; focal severe, chronic, granulomatous and eosinophilic pneumonia with severe, central sclerosis; focal subtotal obstipation of the vessel by the organisation of a precipitation thrombus; multifocal moderate to severe, lympho-plasmacytic, chronic, interstitial pneumonia; multifocal nodular sclerosis (suspicion of obliterated vessels); moderate hyperaemia; moderate to severe, alveolar oedema; multifocal, mild basophilic, plaque-like deposits in the bronchial epithelium; focal, severe, lympho-histiocytic vasculitis with intraluminal nematodes; focal severe, lympho-plasmacellular bronchopneumonia	oligofocal mild, catarrhalic-purulent cholangitis; multifocal moderate, granulomatous and pyo-granulomatous pericholangitis with intralesional eggs of trematodes; severe, follicular, mainly lymphocytic pericholangitis	mild, follicular hyperplasia	moderate hyperaemia	mild, follicular hyperplasia of Peyer patches	severe, diffuse, lympho-plasmocytic tracheitis; multifocal moderate, follicular aggregated, mainly lymphocytic tracheitis; multifocal moderate haemorrhages of the white matter; eye with focal complete separation of the cornea with moderate, non-reactive haemorrhages; mesenterial lymph node with mild, follicular hyperplasia; pulmonal lymph node with severe, follicular hyperplasia; adrenal glands with moderate, non-reactive haemorrhages; skin with follicular complete, non-reactive separation of the epidermis and dermis with mild to moderate haemorrhages; tonsil with mild follicular hyperplasia; within normal limits: aorta, heart, stomach, brain, retropharyngeal lymph node, musculature, diaphragm, pancreas, tongue, thymus, hypophysis

223	yes	moderate, pulmonal endoparasitosis with nematodes in deeper airways and in the parenchyma, partly with associated with lobular restricted subtotal atelectasis; multifocal moderate granulomatous and eosinophilic focal pneumonia; oligofocal mild to moderate, lymphocytic-plasmacellular and eosinophilic bronchitis and peribronchitis; mild alveolar oedema; moderate to severe alveolar and interstitial emphysema	within normal limits (autolysis)	within normal limits (autolysis)	within normal limits	small intestine: oligofocal moderate, perivascular associated, granulomatous and eosinophilic mural enteritis; large intestine: focal moderate, mural hypercellularity (suspicion of mural enteritis; autolysis)	skin: focal severe erosive and purulent acute dermatitis; irregular tissue margin with high amount of bacteria as well as severe auto- and heterolysis; pulmonal lymph node: solitary non-reactive nematode larvae in one intermediate sinus; mesenterial: mild, follicular hyperplasia, high amount of non-reactive bacteria in the sinus of the margin; tonsil: mild follicular hyperplasia; within normal limits: thyroid, retropharyngeal lymph node, musculature, diaphragm, oesophagus, adrenal gland, urinary bladder, umbilical artery, spinal cord (autolysis), eye, tongue, heart, aorta, pancreas

226	no	severe diffuse hyperaemia, mild acute haemorrhage (caused by killing); multifocal mild alveolar emphysema; oligofocal, moderate, chronic, granulomatous pneumonia with participation of single, eosinophilic granulocytes and with intralesional nematodes; suspicion of multifocal, moderate hypertrophy of the tunica media of the pulmonal arteries	within normal limits	moderate, extramedullar haematopoiesis; moderate deposition of pigments in macrophages (suspicion of hemosiderosis)	within normal limits	small intestine: diffuse, mild, lymphocytic plasmacellular and eosiniphilic infiltration of the lamina propria; large intestine: oligofocal, moderate to severe acute erosive colitis with infiltration of neutrophilic and eosinophilic granulocytes and lesion adjacent, intraluminal, metazoan parasitic structures	tonsil: mild, follicular hyperplasia; spinal cord: severe, multifocal, acute haemorrhages (caused by killing); stomach: multifocal, mild, perivascular, lympho-plasmocytic, mural gastritis; pulmonal and hepatic lymphnode: moderate follicular hyperplasia; retropharyngeal lymphnode: severe follicular hyperplasia; mesenterial lymphnode: mild follicular hyperplasia;

227	yes	mild pulmonal endoparasitosis with reactionless nematodes in the alveoli; oligofocal moderate chronic granulomatous pneumonia with participation of eosinophilic granulocytes and with intralesional metazoan parasitic structures; multifocal mild to moderate, peribronchiolar and interstitial, lympho-plasmocytic and eosinophilic as well as neutrophilic pneumonia; suspicion of moderate hypertrophy of the media of pulmonal arteries; moderate diffuse hyperaemia	multifocal mild lympho-histiocytic hepatitis	severe follicular hyperplasia; moderate extramedullar haematopoiesis; mild deposition of pigments in macrophages (suspicion of hemosiderosis); multifocal transcapsular protrusion of red pulpa	within normal limits	small intestine: segmental moderate, diffuse, lympho-plasmocytic enteritis with intraluminal, metazoan parasitic structures; large intestine: focal mild neutrophilic, eosinophilic and histiocytic colitis with intralesional metazoan parasitic structures	pulmonal lymphnode: severe, follicular hyperplasia, blood resorption, mild sinus histiocytosis; mesenterial lymphnode: severe follicular hyperplasia; spinal cord: severe acute haemorrhages, segmental severe axon swelling and haemorrhages and dilatation of the myelin sheath; tonsil: severe follicular hyperplasia
228	yes	severe hyperaemia; multifocal, severe, acute parenchymal haemorrhages; focal, moderate, granulomatous pneumonia with macrophages and giant cells with severely vacuolated cytoplasm (lipid pneumonia)	multifocal, mild, lympho-histiocytic hepatitis	moderate, follicular hyperplasia; moderate, extramedullar haematopoiesis; moderate deposition of pigments in macrophages (suspicion of hemosiderosis)	within normal limits	small intestine: several, intraluminal, metazoan parasitic structures; large intestine: diffuse, mild to moderate lympho-plasmocytic colitis; oligofocal, mild, lympho-plasmocytic, mural colitis	pulmonal lymphnode: severe blood resorption; mesenterial lymphnode: moderate, follicular hyperplasia, mild to moderate, multifocal to confluent, eosinophilic and lympho-histiocytic capsular inflammation; oesophagus: oligofocal, moderate, periglandular, lympho-histiocytic plasma cellular, mural oesophagitis

229	no	severe, diffuse hyperaemia; multifocal, severe, acute parenchymal haemorrhages; focal, mild to moderate, lobular overarching, eosinophilic, alveolar, focal pneumonia with multifocal, mild to moderate, perivascular, interstitial, eosinophilic and lympho-histiocytic infiltration	moderate, hepatocellular deposition of pigment; multifocal, mild to moderate, lymphohistiocytic hepatitis	moderate, extramedullar haematopoiesis; mild deposition of pigment in macrophages (suspicion of hemosiderosis)	within normal limits	small intestine: mild, intraluminal, metazoan parasitic structures; large intestine: focal mild, eosinophilic and lymphohistiocytic, mural colitis	thymus: focal, solitary cyst with flat cell edging; stomach: multifocal to confluent, moderate, lymphohistiocytic, mural gastritis; pulmonal lymphnode: moderate, follicular hyperplasia, moderate anthracosis; mesenterial lymphnode: mild, follicular hyperplasia
230	no	mild pulmonal endoparasitosis with reactionless nematodes in the alveoli; severe hyperaemia; multifocal, severe acute parenchymal haemorrhages; multifocal, mild, lympho-histiocytic, interstitial pneumonia	oligofocal, moderate to severe portal fibrosis with proliferation of the bile duct and partial strangulation of hepatocytic islands; multifocal, mild to moderate, lympho-histiocytic hepatitis	moderate, extramedullar haematopoiesis; moderate deposition of pigments in macrophages (suspicion of hemosiderosis)	within normal limits	small intestine: oligofocal, severe, mural, lympho-histiocytic and granulomatous enteritis with participation of eosinophilc granulocytes; large intestine: severe, intraluminal, metazoan parasitic structures	spinal cord: multifocal, mild, acute haemorrhages; tongue: focal, severe, acute, subepithelial haemorrhage; tonsil: mild, follicular hyperplasia; pulmonal lymph node: mild, follicular hyperplasia; mesenterial lymph node: moderate, follicular hyperplasia; focal, moderate, granulomatous lymphadenitis; multifocal, mild, capsular, lympho-plasmocytic inflammation; retropharyngeal lymphnode: moderate, follicular hyperplasia

231	no	severe, diffuse hyperaemia; multifocal, mild, alveolar emphysema; suspicion of moderate hypertrophia of the media of pulmonal arteries; oligofocal to confluent, mild, lympho-plasmocytic and eosinophilic, interstitial pneumonia associated with mild, eosinophilic arteritis	multifocal, mild to moderate, lympho-histiocytic hepatitis; oligofocal mild, necrotic hepatitis with mainly histiocytic infiltration	mild, extramedullar haematopoiesis	within normal limits	missing	adrenal gland: one side with focal nodular hyperplasia; thyroid: focal severe, mainly lymphocytic thyroiditis; pulmonal and mesenterial lymphnode: severe, follicular hyperplasia; retropharyngeal lymphnode: mild, follicular hyperplasia; spinal cord: single, acute, perivascular haemorrhages
232	yes	mild, pulmonal endoparasitosis with reactionless nematodes in the alveoli; severe, diffuse hyperaemia; severe, acute, confluent haemorrhages of the parenchyma; suspicion of moderate hypertrophy of the media of pulmonal arteries; multifocal, mild, alveolar and interstitial emphysema	multifocal, mild, lympho-histiocytic hepatitis	mild, follicular hyperplasia; moderate, extramedullar haematopoiesis; severe deposition of pigment in macrophages (suspicion of hemosiderosis)	within normal limits	small intestine: mild, intestinal endoparasitosis with metazoan parasitic structures in the lumen; focal, severe, granulomatous and eosinophilc enteritis with intralesional parasitic structures; large intestine: within normal limits	tonsil: severe, follicular hyperplasia and multifocal, moderate, acute, follicular haemorrhages; pulmonal and mesenterial lymph nodes: severe, follicular hyperplasia; stomach: oligofocal, mild, perivascular, lympho-plasmocytic, mural gastritis; spinal cord: multifocal, mild to moderate, acute haemorrhages

233	yes	severe, diffuse hyperaemia; multifocal to confluent severe, acute parenchymal haemorrhages; focal, mild, intraalveolar, osseous metaplasia; oligofocal, mild, mainly lymphocytic, perivascular, interstitial pneumonia; oligofocal mild, eosinophilic, alveolar, focal pneumonia; multifocal, mild, alveolar emphysema	olifofocal, mild, lympho-histiocytic hepatitis	moderate, follicular hyperplasia; moderate, extramedullar haematopoiesis; moderate deposition of pigments in macrophages (suspicion of hemosiderosis)	focal, mild, lympho-plasmacellular, interstitial nephritis	small intestine: focal mild, lympho-histiocytic and eosinophilic, mural enteritis; large intestine; mild, intraluminal metazoan parasitic structures	tonsil: severe, follicular hyperplasia; pulmonal lymphnode: severe resorption of blood, moderate, follicular hyperplasia; mesenteric lymphnode: severe, follicular hyperplasia; spinal cord: mild, acute, leptomeningeal haemorrhages; stomach: multifocal to confluent, mild to moderate, mural, lympho-histiocytic and eosinophilic gastritis
234	yes	severe, diffuse, hyperaemia; multifocal severe, acute parenchymal haemorrhages; multifocal, mild, perivascular, interstitial lympho-plasmocytic pneumonia	oligofocal mild, lymphocytic pericholangitis, partly with mild fibrosis; oligofocal mild, lympho-histiocytic hepatitis	severe deposition of pigments in macrophages (suspicion of hemosiderosis); mild, extramedullar haematopoiesis	within normal limits	small intestine: focal moderate, chronic, lympho-histiocytic and plasmocytic enteritis with central fibrosis; large intestine: multifocal to confluent, mild to moderate, lympho-histiocytic and plasmocytic, partly associated to blood vessels, mural colitis	gall bladder: multifocal, mild, lympho-plasmocytic cholecystitis; spinal cord: severe haemorrhages in the central channel with rupture of the ependyma; mesenterial lymphnode: severe, follicular hyperplasia; pulmonal lymphnode: moderate, follicular hyperplasia; stomach: oligofocal, mild, perivascular, lympho-histiocytic, mural gastritis with mild fibrosis; tonsil: severe, follicular hyperplasia; heart valves: mild, stromal fibrosis

Table S4: Histopathological findings. The table lists the sample IDs, if antimicrobial-resistant *E. coli* were isolated from the sample, and the main histopathological findings for each investigated organ.
