# Supplementary material for: Occurrence of Antimicrobial-Resistant Escherichia coli in Marine Mammals of the North and Baltic Seas: Sentinels for Human Health
Source: Antibiotics (Basel). 2022 Sep 14;11(9):1248. doi: 10.3390/antibiotics11091248 (PMC9495373; doi:10.3390/antibiotics11091248)
Supplement: Supplementary file 1 [file antibiotics-11-01248-s001.zip › Table S5_with change.pdf]

ID	Resistance	Diagnoses	Overall health status	State of nutrition	Age	Sex	Mercy killed	State of decomposition
1	no	unclear	moderate	good	adult	male	yes	poor
56	yes	gastritis, endoparasitosis GIT, otherwise unclear	moderate	good	juvenile	female	no	good
79	no	pulmonar parasitosis	poor	poor	juvenile	female	yes	moderate
80	yes	endoparasitosis with final septicaemia by Streptococcus phocae	poor	poor	juvenile	female	yes	moderate
81	no	pulmonal parasitosis, bronchopneumonia	poor	poor	juvenile	male	yes	moderate
82	yes	premature birth, suspicion of trauma	moderate	not determined - neonate	neonate	female	no	moderate
83	no	suspicion of trauma	moderate	good	juvenile	female	no	moderate
85	no	perinatal death (abandonment)	moderate	good	neonate	female	no	moderate
86	yes	bronchopneumonia, endoparasitosis, cachexia	poor	poor	juvenile	male	no	good
92	yes	potential trauma/sepsis, otherwise unclear	poor	moderate	juvenile	female	no	fresh
93	no	unclear; by-catch cannot be excluded	moderate	good	juvenile	female	no	moderate
130	no	suspicion of by-catch; hepatitis, nephrosis, suspicion of septicaemia by Streptococcus dysgalactiae	poor	good	adult	male	no	moderate
131	no	by-catch; bronchopneumonia	poor	moderate	juvenile	female	no	fresh
132	no	suspicion of by-catch	good	good	juvenile	male	no	fresh
135	no	by-catch; bronchopneumonia, dermatitis, panniculitis	poor	poor	juvenile	female	no	fresh
136	no	by-catch; bronchopneumonia	poor	moderate	adult	male	no	fresh
148	yes	abscess-forming lymphadenitis with final septicaemia due to Hafnia alvei	poor	moderate	juvenile	female	no	poor
169	no	suspicion of septicaemia due to Streptococcus dysgalactiae and β-haemolytic streptococci	moderate	good	juvenile	male	no	moderate
172	yes	bronchopneumonia/pleuritis with fibrosis with final sepsis by Stretococcus dysgalactiae; animal in parturition	poor	moderate	adult	female	no	moderate

193	yes	suspicion of by-catch	good	not determined - neonate	neonate	male	no	fresh
208	yes	suspicion of trauma	good	good	juvenile	male	no	fresh
210	no	hunted	moderate	not indicated	juvenile	female	yes	fresh
211	yes	hunted	moderate	not indicated	juvenile	male	yes	fresh
212	yes	hunted	moderate	not indicated	juvenile	male	yes	fresh
214	yes	hunted	moderate	moderate	adult	female	yes	fresh
215	yes	Septicaemia due to Enterococcus faecalis and E. coli; cachexia	poor	poor	juvenile	female	yes	moderate
216	no	suspicion of trauma	good	moderate	juvenile	male	no	moderate
218	no	suspicion of by-catch; fish remains in the stomach	good	good	neonate	male	no	moderate
221	no	by-catch; bronchopneumonia, cholangitis and pericholangitis	poor	moderate	adult	male	no	fresh
222	yes	by-catch; bronchopneumonia	poor	moderate	adult	male	no	fresh
223	yes	Sepeticaemia due to Streptococcus phocae (portal of entrance head wound/dermatitis or lung/bronchopneumonia)	poor	poor	juvenile	male	yes	good
226	no	shot (hunting quote); oligofocal, moderate, granulomatous pneumonia; erosive colitis	moderate	moderate	juvenile	male	yes	fresh
227	yes	shot (hunting quote); focal, moderate, granulomatous pneumonia; multifocal, mild to moderate peribronchial and interstitial pneumonia; moderate enteritis; focal, mild colitis; hyperplasia of lymphatic tissues	moderate	good	juvenile	female	yes	fresh
228	yes	shot (hunting quote); focal granulomatous pneumonia; mild to moderate colitis; hyperplasia of lymphatic tissues	moderate	good	adult	male	yes	fresh
229	no	shot (hunting quote); pneumonia, gastritis, colitis, hepatitis, moderate endoparasitosis	moderate	good	adult	male	yes	fresh

230	no	shot (hunting quote); enteritis; portal fibrosis of the liver; mild to moderate hepatitis; mild interstitial pneumonia	moderate	good	adult	female	yes	fresh
231	no	shot (hunting quote); pneumonia, hepatitis, thyreoiditis, hyperplasia of lymphatic tissues	moderate	good	juvenile	female	yes	fresh
232	yes	shot (hunting quote); enteritis; hyperplasia of lymphatic tissues	moderate	good	adult	female	yes	fresh
233	yes	shot (hunting quote); enteritis; pneumonia, gastritis; hyperplasia of lymphatic tissues	moderate	good	adult	female	yes	fresh
234	yes	shot (hunting quote); enteritis; colitis; hyperplasia of lymphatic tissues	moderate	moderate	adult	male	yes	fresh
235	no	shot (hunting quote); erosive colitis; mural colitis; hepatitis; hyperplasia of lymphatic tissues	moderate	good	juvenile	female	yes	fresh

Table S5: Main diagnoses and general information. The table lists the sample IDs, if antimicrobial-resistant E. coli were isolated from the sample, the main diagnoses, a classification of the overall health status per animal, the state of nutrition, the age, the sex, if the animal was mercy killed or died on its own, and the state of decomposition.
